# Unveiling the nature of supramolecular crown ether–C_60_ interactions†
†This work is dedicated to Professor José Barluenga on the occasion of his 75th birthday.
‡
‡Electronic supplementary information (ESI) available: Experimental procedures, titration experiments, MS spectra, electrochemistry experiments, transient absorption measurements and computational details. See DOI: 10.1039/c5sc00850f



**DOI:** 10.1039/c5sc00850f

**Published:** 2015-05-18

**Authors:** Luis Moreira, Joaquín Calbo, Rafael M. Krick Calderon, José Santos, Beatriz M. Illescas, Juan Aragó, Jean-François Nierengarten, Dirk M. Guldi, Enrique Ortí, Nazario Martín

**Affiliations:** a Departamento de Química Orgánica , Facultad de Química , Universidad Complutense de Madrid , 28040 Madrid , Spain . Email: nazmar@ucm.es; b Instituto de Ciencia Molecular , Universidad de Valencia , 46980 Paterna , Spain . Email: enrique.orti@uv.es; c Department Chemie und Pharmazie , Friedrich-Alexander-Universität , 91058 Erlangen , Germany . Email: dirk.guldi@fau.de; d Laboratoire de Chimie des Matériaux Moléculaires , Université de Strasbourg et CNRS (UMR 7509) , 67087 Strasbourg , France . Email: nierengarten@unistra.fr

## Abstract

A series of exTTF-(crown ether)_2_ receptors, designed to host C_60_, has been prepared. The size of the crown ether and the nature of the heteroatoms have been systematically changed to fine tune the association constants. Electrochemical measurements and transient absorption spectroscopy assisted in corroborating charge transfer in the ground state and in the excited state, leading to the formation of radical ion pairs featuring lifetimes in the range from 12 to 21 ps. To rationalize the nature of the exTTF-(crown ether)_2_·C_60_ stabilizing interactions, theoretical calculations have been carried out, suggesting a synergetic interplay of donor–acceptor, π–π, n–π and CH···π interactions, which is the basis for the affinity of our novel receptors towards C_60_.

## Abstract 2

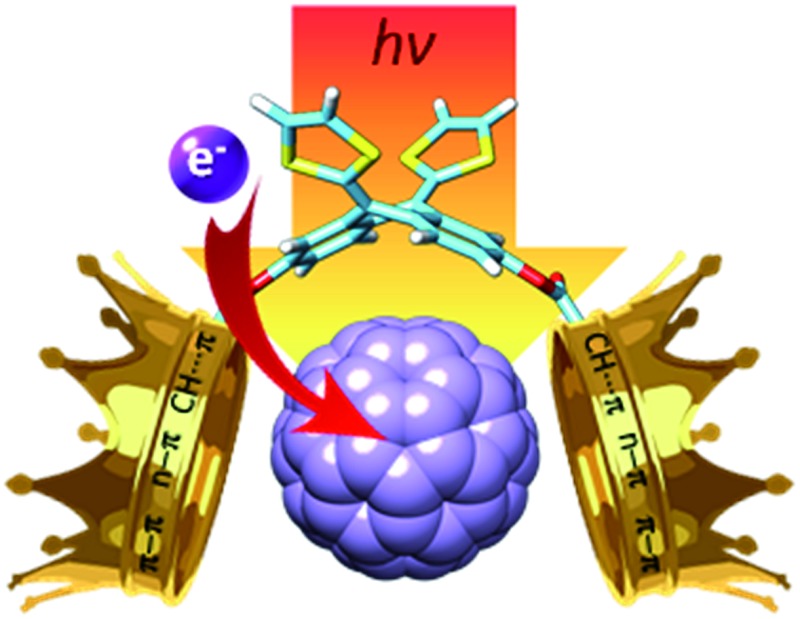
Preparation of exTTF-(crown ether)_2_ receptors, which host C_60_, to understand the nature of the fullerene–crown ether interaction. A combination of experimental and *in silico* studies suggest that it results from the interplay of donor–acceptor, ð–ð , n–ð and CH•••ð interactions.

## Introduction

The construction of non-covalent electron donor–acceptor (D–A) assemblies is a rational way for the creation of new and sophisticated electroactive materials that are impossible to obtain by covalent means. As representative examples, a variety of simple models for the study of electron and energy transfer processes, such as those found in the photosynthetic centers of plants and bacteria, have been prepared.^1,2^ To this end, fullerenes have been widely used as electron acceptors given their spherical geometry,^3^ small reorganization energy in electron transfer reactions,^4^ low reduction potential,^5,6^ appreciable absorption cross section throughout a wide range of the solar spectrum,^7^ and efficient generation of long-lived charge-separated states following photoexcitation.^8–10^ In this regard, their supramolecular chemistry is largely based on the use of fullerene derivatives, giving rise to metal–ligand interactions, π–π stacking, hydrogen bonding, electrostatic interactions, or mechanical bonds.^11–16^ This approach involves, however, saturating at least one of their double bonds, thus altering their electronic conjugation. In contrast, the complexation of pristine fullerene enables their singular electronic properties to be maintained. This is typically achieved by using host molecules endowed with large dispersion forces, namely π–π and van der Waals interactions, such as cyclodextrins,^17–19^ calixarenes,^20–22^ cyclotriveratrylenes,^23–25^ porphyrins,^26–30^ subphthalocyanines,^31–33^ or 9,10-di(1,3-dithiol-2-ylidene)-9,10-dihydroanthracenes (exTTF).^34–37^

Interestingly, although an aza-crown ether decorated with lipophilic fragments was the first system ever reported to complex fullerenes in solution,^38^ crown ethers have scarcely been explored as hosts for fullerenes. Indeed, despite the importance of both kinds of molecules, whose discoverers were each awarded a Nobel Prize,^39,40^ to the best of our knowledge, a detailed experimental and theoretical study on the supramolecular interactions occurring between them has not been properly addressed thus far and many open questions still remain unanswered. A notable exception includes the work by Mukherjee and co-workers,^41,42^ who observed that the overall stability of the resulting complexes increased as a function of the cavity size of the crown ether. To this end, an interplay between different energy terms, such as solvation effects, electron donor–acceptor interactions, *etc.*, was hypothesized to explain the complexation. Following the latter, Liu *et al.* evaluated the impact of introducing Se atoms into the crown ethers, which led to a better stabilization.^43^ As in the previous example, a relation between the cavity size of the crown ether and the binding constants with C_60_ was noted. Another remarkable example is the porphyrin designed by D'Souza *et al.*, bearing four benzo[18]crown-6 ethers in the *meso* positions and exhibiting a moderate but tunable affinity towards C_60_ depending on the presence or the absence of K^+^ ions.^44^ More recently, our group developed a novel receptor for C_60_, which was based on an exTTF derivative appended with two benzo[18]crown-6 ethers, exTTF-(crown ether)_2_. This receptor featured extraordinarily high binding constants (*K*_a_) for C_60_ and C_70_ with log *K*_a_ = 6.7 and 7.4 in benzonitrile at room temperature, respectively. As a matter of fact, it became the sole example of C_60_ complexation by a single exTTF molecule.^45^

The impact of crown ethers on the receptor properties led us to focus on the study of the crown ether·C_60_ interaction in order to finally unveil its nature. To do so, a series of exTTF-crown ether derivatives have been prepared, in which the cavity size and the nature of the heteroatoms have been systematically modified. Their complexation with C_60_ has been complementarily investigated by both spectroscopic and electrochemical means. In addition, theoretical calculations have been carried out to draw conclusions about the key factors influencing the resulting binding constants.

## Results and discussion

**Scheme 1:**
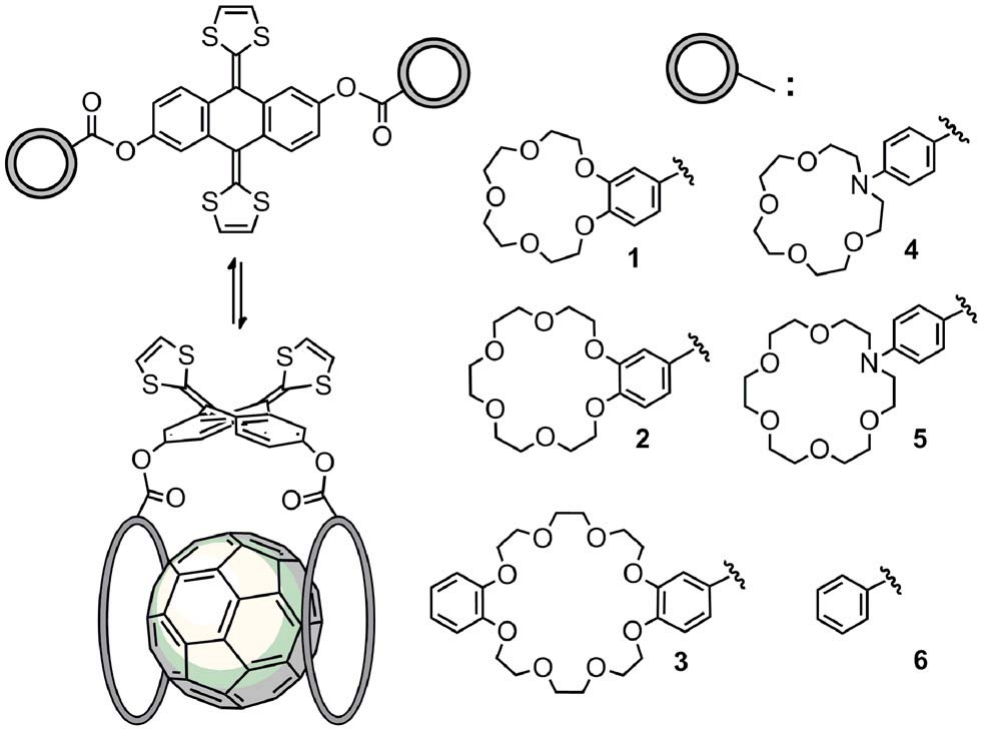
Complexes obtained from exTTF-based **1–6** and C_60_ at rt in PhCl.

In order to carry out this study, we have designed and synthesized a series of novel exTTF-based receptors endowed with two crown ethers, **1–5** (Scheme 1). Non-commercially available crown ethers were obtained through a Buchwald–Hartwig cross coupling reaction or a Williamson ether synthesis (see ESI‡ for further details). Then, 9,10-bis(1,3-dithiol-2-ylidene)-9,10-dihydroanthracene-2,6-diol^46^ was esterified with the corresponding crown ether appended carboxylic acids either *via* the acyl chloride or activation with EDC. Compound **6**, lacking the crown ether moieties, was also prepared as a reference by condensation of the 2,6-dihydroxylated exTTF with benzoic acid.

The unambiguous characterization of all compounds was carried out by employing standard spectroscopic and analytical techniques (see ESI‡ for details on the synthesis and characterization). Successful esterification was evidenced by the maintenance of the characteristic 1,3-thiol signal at around 6 ppm and the appearance of the crown ether signals in the 3–4 ppm region of the ^1^H-NMR spectra. The UV-vis spectroscopy profiles of all exTTFs exhibited the characteristic band at around 435 nm without any significant shifts among the derivatives.

### Titration experiments

**Fig. 1:**
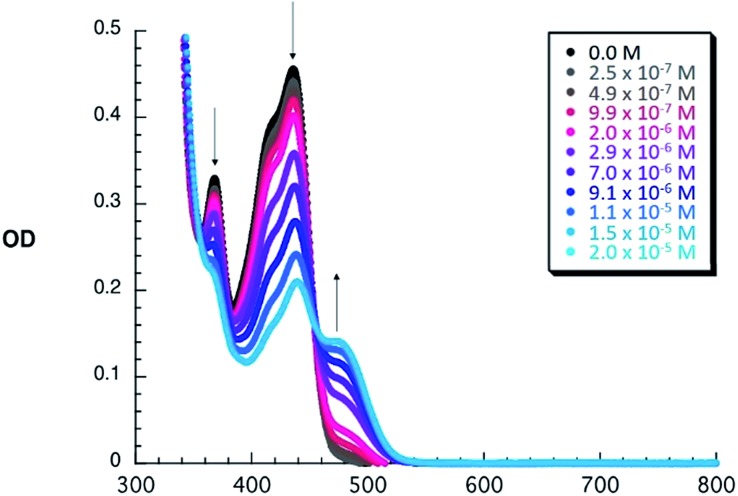
Absorption spectra of dilute PhCl solutions of **3** (1.5 × 10^–5^ M) with variable concentrations of C_60_ upon subtraction of the fullerene absorption profile to highlight the absorption changes and the isosbestic point.

To shed light on the ground state interactions between **1–6** and C_60_, absorption titrations were performed in PhCl at room temperature (see ESI‡ for further details). When adding C_60_ to constant concentrations of **1–6**, an increase of the typical absorption features of C_60_ evolve, namely a strong absorption band at wavelengths < 350 nm, a sharp band at 407 nm, and a broad absorption between 470 and 650 nm. Simultaneously, the intrinsic exTTF features, which are noted between 350 and 450 nm, gradually decrease in the presence of C_60_. All of these changes are assigned to a successful complexation of C_60_ by **1–6**. Additional support for this notion comes from a newly developing absorption band, which features a C_60_^*δ*–^/exTTF^*δ*+^ charge transfer character, between 455 and 530 nm – peaking at 475 nm – in PhCl. When going to the more polar PhCN, a shift of the charge transfer band is observed (455–550 nm, peaking at 485 nm). This spectral shift is rationalized on grounds of a better stabilization of C_60_^*δ*–^/exTTF^*δ*+^ (see Fig. S2‡). This charge transfer interaction has also been observed in other donor–C_60_ systems providing a moderate contribution to the overall stability of exTTF based fullerene receptors.^47^ As a representative example, the spectroscopic changes observed for **3** upon titration with C_60_ are depicted in Fig. 1. These spectral changes are a clear signature for the association of **3** with C_60_ and were also observed for all other receptors (Fig. S1 and S2 in the ESI‡).

The binding constants of **1–6** with C_60_ were obtained with non-linear curve fitting analyses of the UV-vis absorption titration experiments. For all the studied systems, the best fits to the experimental data were obtained when assuming a 1 : 1 stoichiometry, which was also observed by MS-MALDI experiments (Fig. S4‡). As documented in Table 1, the nature and size of the crown ether has a clear impact on the affinity towards C_60_, leading to *K*_a_ values that vary by as much as three orders of magnitude. A clear trend between the size of the crown ether and the magnitude of the binding constant can be obtained in PhCl. Thus, the highest constant of the series is obtained for **3**, bearing the largest crown ether. The lowest binding constant is obtained for reference compound **6**, lacking the crown ether subunits. In between these, both bis-aza-crown ether derivatives **4** and **5** exhibit significantly smaller *K*_a_ values when compared to their corresponding oxygen-bearing analogues **1** and **2**, respectively. This experimental finding could be accounted for by their less appropriate geometry to accommodate C_60_, as will be further discussed in the computational studies (see below).

**Table 1:** Calculated binding constants (log *K*_a_) for exTTF molecular tweezers **1–6** towards C_60_ in PhCl at 298 K

**1·C_60_**	**2·C_60_**	**3·C_60_**	**4·C_60_**	**5·C_60_**	**6·C_60_**
4.8 ± 0.9	6.7 ± 0.2 (ref. 45)	6.9 ± 0.2	3.8 ± 0.6	5.1 ± 0.1	3.3 ± 0.4

Insights into the excited state interactions between **1–6** and C_60_ in either PhCl or PhCN came from emission studies, exciting at 350, 400, and 450 nm (see ESI‡). Upon the addition of C_60_ to a solution of **1–6**, a new and broad emission at around 530 and 550 nm in PhCl and PhCN, respectively, grows at the expense of the intrinsic exTTF emission centered around 460 nm. The substantial 530 to 550 nm shift is due to the underlying intermolecular charge transfer character. Like in the ground state, the more polar PhCN facilitates the stabilization of the (C_60_^*δ*–^/exTTF^*δ*+^)* excited state, when compared to PhCl (see Fig. S3‡). Importantly, the underlying energetics are, on one hand, comparable to those found for other C_60_/exTTF systems, and, on the other hand, appreciably higher than what is typically found in C_60_/porphyrins, with values of 2.2 and 1.5 eV, respectively.^48–53^

### Electrochemical studies

**Fig. 2:**
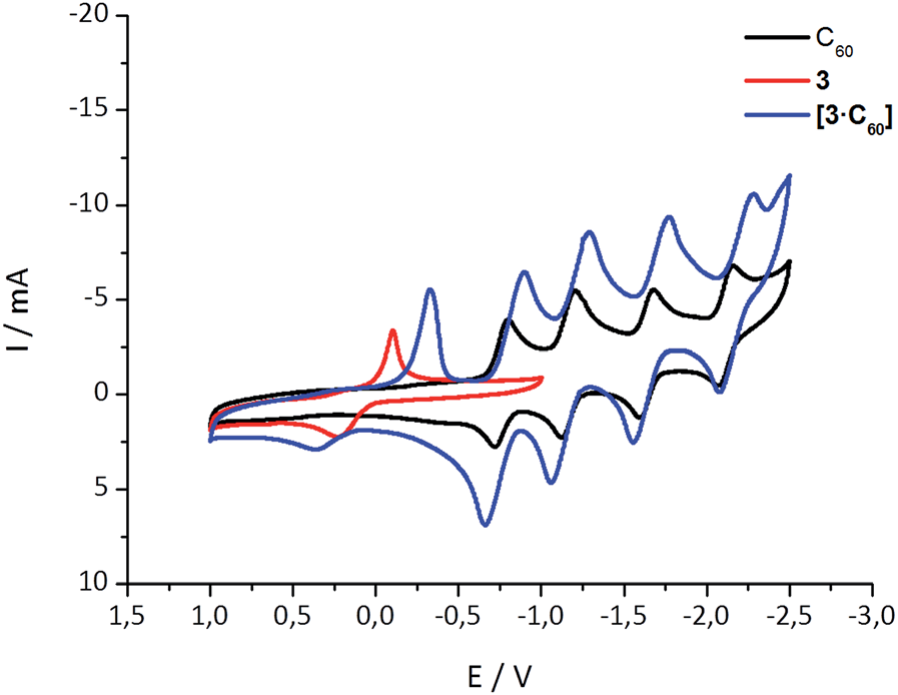
Cyclic voltammetry of **3**, C_60_, and **3·C_60_** in a 4/1 v/v solvent mixture of PhCl and MeCN with 0.1 M *n*-Bu_4_NPF_6_ and at 0.1 V s^–1^. Potentials are given *versus* Ag/Ag^+^.

Further insights into the interactions in the ground state came from cyclic voltammetry (CV) investigations with equimolar mixtures of **1–6** and C_60_. Notably, the lower stability of the aza-crown ether complexes limited the electrochemical measurements to the crown ethers as summarized in Table S1.‡ As an example, Fig. 2 shows the CVs for C_60_, **3**, and a 1 : 1 mixture of **3** and C_60_ (see ESI‡ for more details). Because of the complexation, the first, second, third, and fourth quasireversible reductions of C_60_ are clearly shifted to more cathodic potentials (see Table S1 and Fig. S5‡). The smallest shift for the first reduction (∼30 mV) is found for **6·C_60_**, it increases to ∼50 mV for both **1·C_60_** and **2·C_60_**, and to ∼100 mV for **3·C_60_**. At this point, we postulate that the magnitude of the reduction potential shift, as seen upon complexation, relates to the binding strength. The electronic interactions between the electron-donating exTTF host and the electron-accepting C_60_ guest in the ground state are the basis for this trend and they scale with the size of the crown ether. A shift is also found in the exTTF-centered oxidation, moving towards more positive values (see Fig. 2 and S5‡). These shifts, however, do not fully correlate with the experimentally determined binding constants, most likely due to adsorption phenomena upon oxidation.

### Transient absorption studies

In the transient absorption measurements with **1–5** in PhCl, only a single transient state evolves. The excited state transient, which is exTTF-centered, appears simultaneously with the conclusion of the 387 nm laser excitation. Transient maxima arise at 465, 605, and 910 nm, while ground state bleaching is observed at wavelengths < 450 nm. Kinetic analyses of the transient states reveal short-lived excited states with lifetimes in the range of 0.9 to 1.1 ps. Such short lifetimes are rationalized on the basis of strong second-order vibronic spin–orbit coupling, which originates from the sulfur atoms.

With respect to C_60_, upon excitation at 480 nm in PhCl, the characteristic singlet excited state transient emerges in the near infrared around 980 nm. This singlet excited state deactivates *via* intersystem crossing within 1.2 ns and produces the corresponding triplet excited state with a transient maximum at 750 nm and a lifetime of about 45 μs.

**Fig. 3:**
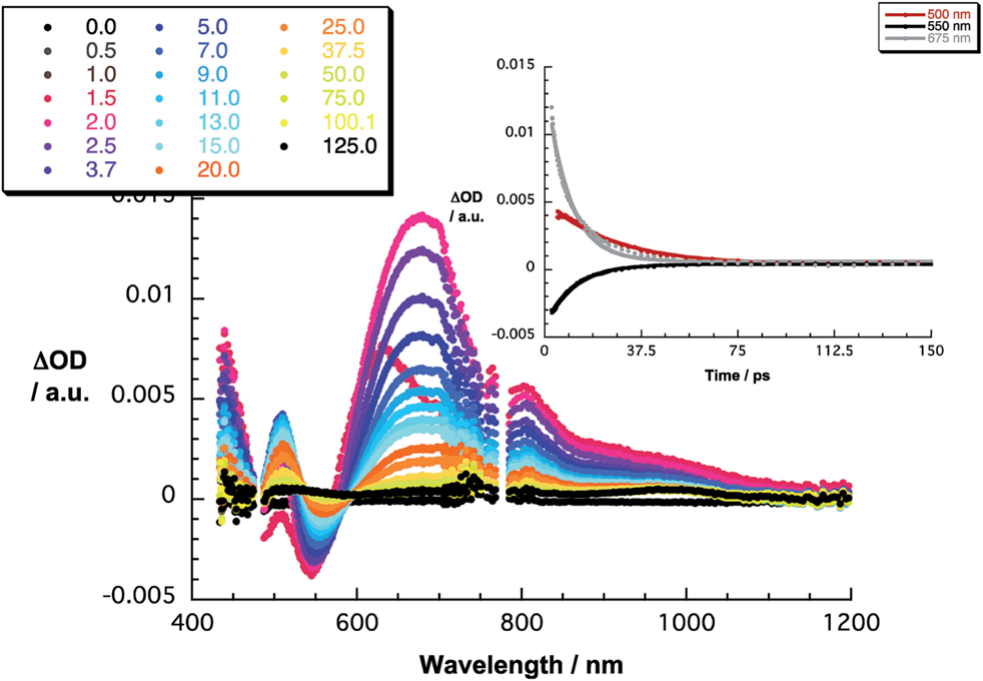
Differential absorption spectra (visible and near-infrared) obtained upon femtosecond flash photolysis (480 nm) of **1·C_60_** (1 : 1) in PhCl [(C_60_/exTTF) ≈ 10^–5^ M] with several time delays between 0 and 125 ps at room temperature. Inset: time–absorption profiles of the spectra at 500, 550, and 675 nm monitoring the charge separation/charge recombination.

The 480 nm excitation of equimolar mixtures of **1–5** and C_60_ [(C_60_/exTTF) ≈ 10^–5^ M] into the charge transfer band results in the instantaneous formation of photoexcited C_60_^*δ*–^/exTTF^*δ*+^, as seen in Fig. 3 for **1·C_60_** and in Fig. S6‡ for the remaining complexes. This excited charge transfer state features maxima at 507 and 673 nm as well as a broad band in the near infrared around 950 nm. Additionally, transient bleaching is observed at around 550 nm. The latter relates, however, to stimulated charge transfer emission – *vide supra*. In terms of kinetics, the transient states transform to the fully charge-separated state, that is, C_60_˙^–^/exTTF˙^+^, on a time scale ranging from 1.6 to 2.3 ps (Table 2). In terms of spectroscopy, the presence of the characteristic transient absorption due to the one-electron oxidized exTTF in the visible region at around 680 nm confirms our hypothesis.^54–56^ Importantly, the latter is complemented by the feature of the one-electron reduced C_60_, which maximizes in the near-infrared at around 1100 nm.^57^ These radical ion pair states recombine in each of the probed systems within 12 to 21 ps into lower lying excited states of C_60_, that is, the singlet and triplet excited states with maxima at 750 and 980 nm, respectively (Table 2). In general, stronger binding causes acceleration of the charge recombination – **3** *vs.* **2** and **5** *vs.* **4** – due to tighter interactions.

**Table 2:** Charge separation (CS) and charge recombination (CR) dynamics obtained upon laser flash photolysis at 480 nm of equimolar mixtures of **1–5** with C_60_ in argon-saturated PhCl

Complex	CS (s^–1^)	CR (s^–1^)
**1·C_60_**	6.3 × 10^11^	8.3 × 10^10^
**2·C_60_**	4.3 × 10^11^	4.8 × 10^10^
**3·C_60_**	4.8 × 10^11^	5.7 × 10^10^
**4·C_60_**	4.3 × 10^11^	7.6 × 10^10^
**5·C_60_**	4.8 × 10^11^	7.9 × 10^10^

### Computational studies

**Fig. 4:**
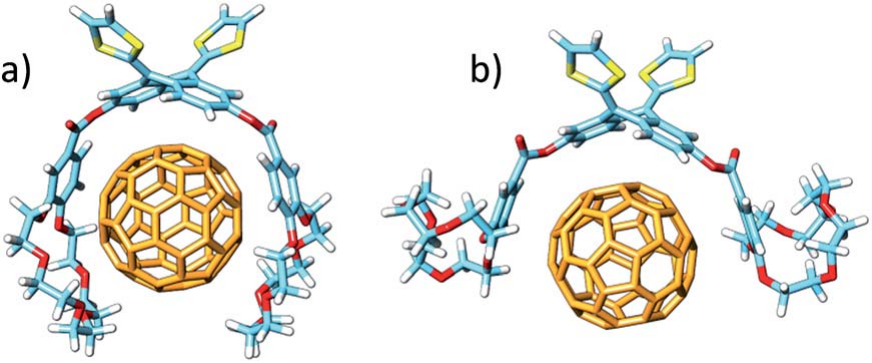
Minimum-energy embraced (a) and non-embraced (b) conformation calculated at the PM7 level for the **2·C_60_** complex.

The different conformations that **1–6** may adopt when complexing C_60_ were initially explored by using semiempirical PM7 calculations. Only 1 : 1 stoichiometric ratios were computed according to the experimental evidences. Fig. 4a shows the minimum-energy optimized structure calculated for **2·C_60_** as a representative example (see Fig. S7‡ for all the associates). In each of the complexes between **1–6** and C_60_, the latter interacts with the anthracene concave region of exTTF and, at the same time, the crown ether-based arms embrace C_60_ with a pinzer-like shape. Non-embraced host–guest arrangements, in which the crown ethers fold themselves away from C_60_, were also optimized for **1·C_60_**, **2·C_60_** and **3·C_60_** (see Fig. 4b for **2·C_60_**) to assess the stabilization due to the embracing movement. PM7 predicts association energies of –68.12, –72.43 and –88.75 kcal mol^–1^ for the embraced conformations of **1·C_60_**, **2·C_60_** and **3·C_60_**, respectively, whereas the values for their non-embraced homologues were computed to be –50.49, –51.20 and –51.56 kcal mol^–1^. The calculations therefore suggest that the embraced conformations are favored by an increase in the total binding energy that increases with the size of the crown ether. Intermediate structures in which C_60_ is embraced by only one arm of the exTTF-(crown ether)_2_ receptor were also calculated for complexes **2·C_60_** and **3·C_60_**. For **2·C_60_**, the two crown ether arms stabilize the complex by a similar amount of energy, about –10.5 kcal mol^–1^ (Fig. S8a‡). In contrast, the first arm of **3·C_60_** stabilizes the complex by –24.9 kcal mol^–1^ due to the larger size of the crown ether and to the additional interaction with the terminal benzene ring, whereas the second arm leads to a significantly lower stabilization of –12.3 kcal mol^–1^ due to the steric hindrance between the two crown ether arms (Fig. S8b‡).

**Fig. 5:**
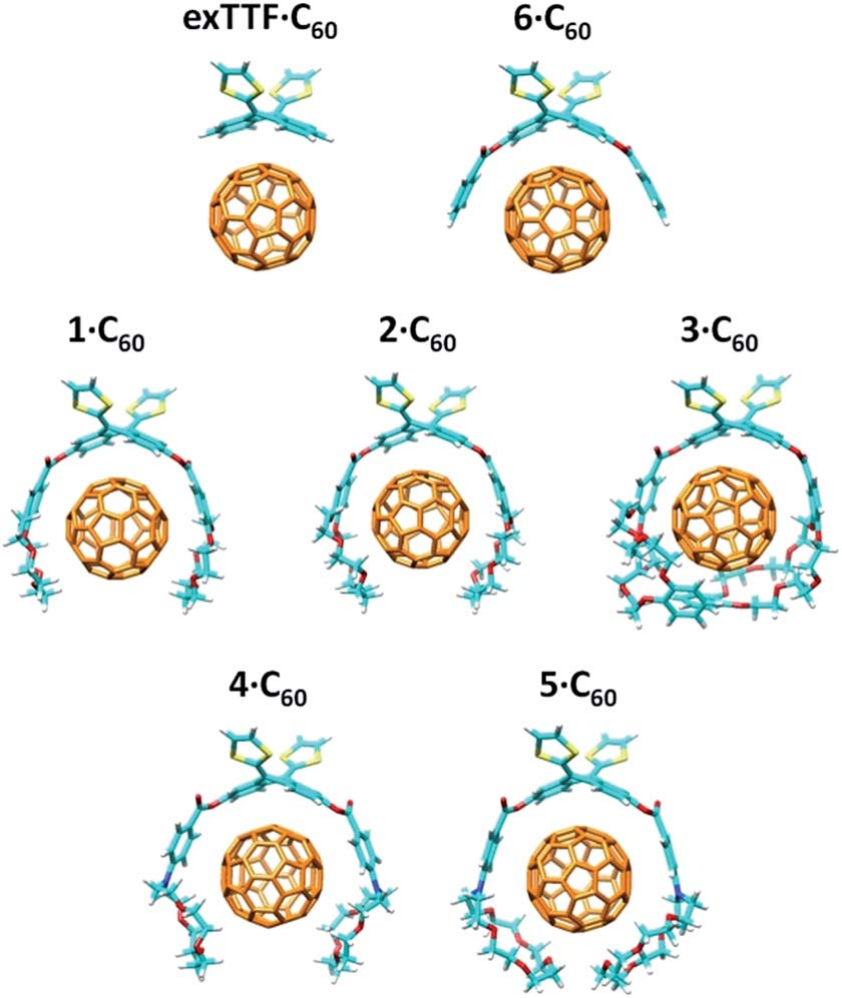
B97-D/cc-pVDZ minimum-energy geometries calculated for the **exTTF·C_60_** and **1–6·C_60_** complexes.

The more stable embraced conformations were subsequently optimized using the dispersion-corrected B97-D functional and the cc-pVDZ basis set (Fig. 5). The **exTTF·C_60_** complex, which is not observed experimentally, was also calculated as a reference. Geometry optimizations were performed under *C*_2_ symmetry restrictions, except for **3·C_60_**. For the latter, no symmetry was assumed because the terminal benzene rings of the crown ethers disturb each other when complexing C_60_, resulting in a *C*_1_ symmetry.

The B97-D/cc-pVDZ-optimized geometries reveal intermolecular contacts of different natures along the host–guest interface. Table 3 summarizes the shortest distances computed for the intermolecular contacts which determine the stabilization of the complexes between **1–6** and C_60_. To estimate the binding energies, single-point energy calculations were performed on the B97-D/cc-pVDZ-optimized structures using the revPBE0-D3 functional and the more extended triple-*ζ* cc-pVTZ basis set. Table 3 gives the binding energies computed for the resulting complexes. A binding energy of –10.24 kcal mol^–1^ is predicted for **exTTF·C_60_** due to the π–π interactions between the lateral benzene rings of exTTF and the benzene rings of C_60_ with centroid–centroid distances of 3.42 Å (*a* in Table 3). Since **exTTF·C_60_** is not detected experimentally, entropic and solvent effects are expected to provide a positive contribution that cancels out the stabilizing interaction. In **6·C_60_**, two additional interactions originating from the presence of the benzoates are found: π–π interactions at 3.25 Å between the benzene rings of the benzoate moieties and C_60_ (*b* in Table 3) and n–π interactions due to short O(host)···C(guest) intermolecular distances (3.16 Å, *c* in Table 3). The positive effect of these interactions is evidenced by the folding angle of the anthracene in exTTF, which becomes sharper in passing from **exTTF·C_60_** (142.5°) to **6·C_60_** (137.0°). The association energy computed for **6·C_60_** amounts to –22.85 kcal mol^–1^, which is more than twice the binding energy found for **exTTF·C_60_**, and, in turn, is high enough to experimentally detect the complex in solution (Table 1).

**Table 3:** Intermolecular distances (*a*–*f*, in Å) and binding energies (*E*_bind_, in kcal mol^–1^) calculated at the B97-D/cc-pVDZ and revPBE0-D3/cc-pVTZ levels, respectively, for the **exTTF·C_60_** and **1–6·C_60_** complexes^*a*^

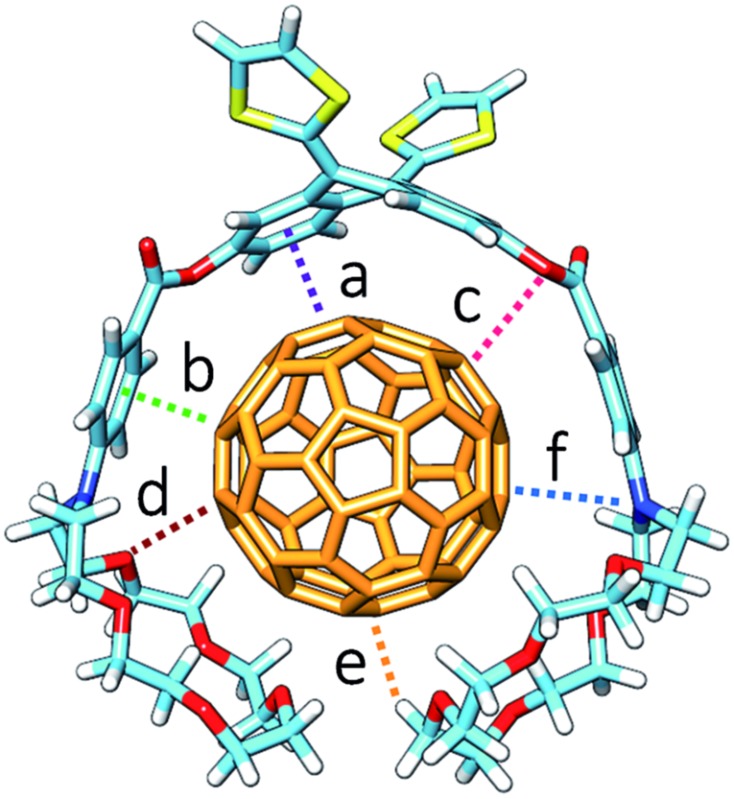							
Complex	*a*	*b*	*c*	*d*	*e*	*f*	*E*_bind_ (kcal mol^–1^)
**exTTF·C_60_**	3.42	—	—	—	—	—	–10.24
**1·C_60_**	3.46	2.99	3.30	3.19	2.61	—	–39.69
**2·C_60_**	3.45	2.95	3.42	2.79	2.69	—	–44.76
**3·C_60_** ^*b*^	3.49^*c*^	2.98^*c*^	3.44^*c*^	2.85	2.50	—	–54.36
**4·C_60_**	3.37	3.41	3.25	3.56	2.57	4.14	–36.77
**5·C_60_**	3.37	3.06	3.14	3.14	2.54	3.50	–43.33
**6·C_60_**	3.45	3.25	3.16	—	—	—	–22.85

^*a*^ *a* is the distance between the centroid of the lateral benzene rings of exTTF and that of the closest benzene rings of C_60_. *b* is the distance between the centroid of the benzene ring of the benzoate moiety and the center of the closest C_60_ 6 : 6 double bond. *c* is the distance between the benzoate sp^3^ oxygen and the closest carbon atom of C_60_. *d* and *e* are the shortest O···C_60_ and H···C_60_ distances, respectively, between the crown ether and C_60_. *f* is the distance between the nitrogen atom of the aza-crown ether and the closest carbon atom of C_60_.

^*b*^Two additional π–π interactions between the outer benzene rings of the crown ethers and C_60_ are computed at 3.13 and 3.68 Å.

^*c*^Average values.

Upon going from **6·C_60_** to **1·C_60_**, **2·C_60_**, and **3·C_60_**, new n–π (*d*) and CH···π (*e*) interactions with intermolecular distances of 3.4 and 2.8 Å (averaged over all the O···C_60_ and C–H···C_60_ interactions shorter than 3.8 and 3.2 Å, respectively, in **1–3·C_60_** associates) contribute to the complex stabilization due to the inclusion of the crown ethers in the host system (Table 3). The calculations predict that the binding energies of the complexes rise as the size of the crown ether increases, passing from –39.69 kcal mol^–1^ for **1·C_60_**, to –44.76 kcal mol^–1^ for **2·C_60_**, and to –54.36 kcal mol^–1^ for **3·C_60_**. This trend is in good agreement with the increase of the *K*_a_ value estimated experimentally (Table 1). It has to be attributed to increasing contributions from the n–π and CH···π interactions, which are associated with the increasing size of the crown ethers when going from **1·C_60_** to **3·C_60_**. The crown ether arms wrap C_60_ and lead to more compact complexes, in which the benzene rings of the benzoate moiety are closer (by 0.2 Å) to C_60_ as compared, for example, with **6·C_60_** (distance *b* in Table 3). This gain in compactness underpins the positive effect that the noncovalent interactions between C_60_ and the crown ethers exert on the complex stability.

**Fig. 6:**
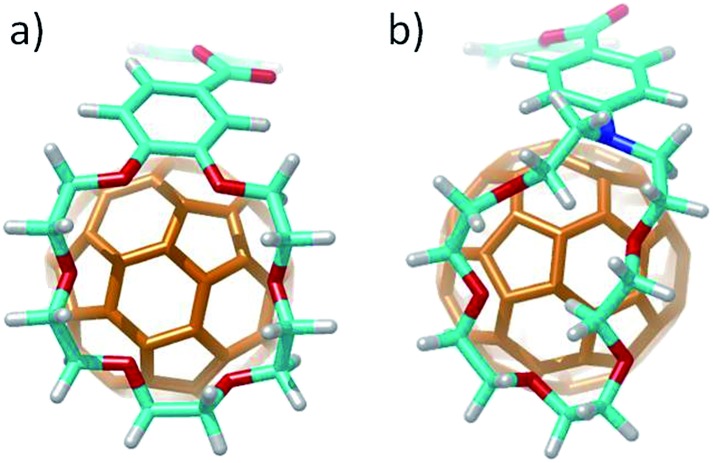
Side view of the B97-D/cc-pVDZ-optimized geometries calculated for complexes **2·C_60_** (a) and **5·C_60_** (b) showing the different spatial arrangement of the crown and aza-crown ethers, respectively, along the C_60_ guest.

Finally, the nitrogen atoms, which bridge the crown ether and the benzoate in **4** and **5**, confer additional flexibility to the aza-crown ethers in **4·C_60_** and **5·C_60_**. The latter features structures which are more folded than their oxygenated analogues **1·C_60_** and **2·C_60_** (Fig. 6). These structures are less appropriate to accommodate C_60_ and, as a consequence, they lead to less efficient host–guest interactions. For instance, in **4·C_60_**, the intermolecular contacts defined by parameters *b* and *d* are found at significantly larger distances relative to **1·C_60_** (Table 3). The binding energies computed for **4·C_60_** (–36.77 kcal mol^–1^) and **5·C_60_** (–43.33 kcal mol^–1^) are indeed smaller than those computed for the oxygenated complexes **1·C_60_** and **2·C_60_** (–39.69 and –44.76 kcal mol^–1^, respectively). The lower affinity, in terms of the interaction with C_60_, for the aza-crown ethers is in agreement with the experimentally determined binding constants (Table 1) and is ascribed to an overall weakening of the host–guest interactions provoked by the less efficiently oriented aza-crown ether arms. The calculations therefore suggest that the ability of the exTTF-based molecular tweezers to bind C_60_ arises from an interplay of different π–π, n–π and CH···π interactions, and that the size and nature of the crown ether are key factors for the relative stabilization of the resulting complexes between **1–6** and C_60_.

The theoretical calculations predict a charge transfer from exTTF to C_60_ for all the complexes in the ground state. For **6·C_60_**, a small charge-transfer of 0.06*e* is computed, which accounts for the negative shift of ∼30 mV observed in the first reduction of **6·C_60_** when compared to C_60_ (Table S1‡). **1–3** interact more strongly with C_60_ and a noticeable increase in the charge transferred to C_60_ is obtained along the series: **1·C_60_** (0.14*e*), **2·C_60_** (0.15*e*), and **3·C_60_** (0.18*e*). Such an increase justifies the larger cathodic shifts measured for the first reductions in **1·C_60_** and **2·C_60_** (∼50 mV), as well as in **3·C_60_** (∼100 mV), when compared to C_60_ (Table S1‡). In the excited state, electron promotion from the HOMO to the LUMO, which are respectively localized on exTTF and C_60_ (Fig. S9‡), leads to a fully charge-separated C_60_˙^–^/exTTF˙^+^ associate.

## Conclusions

In summary, we have obtained a series of new exTTF-(crown ether)_2_ receptors featuring two crown ethers of different size and composition. These receptors bind C_60_ in PhCl with moderate to high efficiencies. The interactions between **1–6** and C_60_ have been further studied both in the ground state by CV, pointing to the presence of electronic interactions, and in the excited state by transient absorption studies. Importantly, the latter corroborates the formation of radical ion pair states, which feature lifetimes in the 12–21 ps range. Complementary computational investigations have further documented the stabilization energy associated with the embraced conformation (25–30%) and have provided critical insights into each of the interactions involved in the process. The nature of the supramolecular exTTF-(crown ether)_2_·C_60_ affinity interactions arises, thus, from an interplay of donor–acceptor interactions and π–π, n–π and CH···π forces whose intensity depends on the size and nature of the crown ether.
