# Supplementary material for: Unveiling the nature of supramolecular crown ether–C60 interactions
Source: Chem Sci. 2015 May 18;6(8):4426–32. doi: 10.1039/c5sc00850f (PMC5665100; doi:10.1039/c5sc00850f)
Supplement: Supplementary file 1 [file SC-006-C5SC00850F-s001.pdf]

Electronic Supplementary Material (ESI) for Chemical Science.
This journal is © The Royal Society of Chemistry 2015

Supporting Information

for

Unveiling the Nature of Supramolecular Crown Ether-C_{60} interactions

Luis Moreira,† Joaquín Calbo,‡ Rafael M. Krick Calderon,§ José Santos,† Beatriz M. Illescas,† Juan Aragó,‡ Jean-François Nierengarten,*‖ Dirk M. Guldi,*§ Enrique Ortí,*‡ Nazario Martín*†

† Departamento de Química Orgánica, Facultad de Química, Universidad Complutense de Madrid, 28040 Madrid, Spain

‡ Instituto de Ciencia Molecular, Universidad de Valencia, 46890 Paterna, Spain

§ Department Chemie und Pharmazie, Friedrich-Alexander-Universität Erlangen/Nürnberg, Egerlandstr. 3, 91058 Erlangen, Germany

‖ Université de Strasbourg et CNRS, ECPM, 25 rue Becquerel, 67087 Strasbourg, Cedex 2, France

Contents

1. General Methods....2

2. General Synthetic Scheme....3

3. Synthesis....4

4. Titration Experiments....9

5. Complementary spectroscopical analysis....12

6. MS Spectra of Supramolecular Complexes with C_{60}....15

7. Electrochemistry....17

8. Transient Absorption Studies....19

9. Theoretical Calculations....20

10. References....24

1. General Methods

Reagents were used as purchased from commercial sources without further purification. Solvents were dried and distilled using standard techniques prior to use.[1] Compounds **2**,[2] **9**,[3],[4],[5] and **15**[6] were prepared according to previously reported procedures. All reactions were performed in standard glassware under an inert Ar atmosphere. Analytical thin-layer chromatography was performed using aluminum-coated Merck Kieselgel 60 F254. Visualization was made by UV light or I_2 vapor. Purification of crude reaction mixture was achieved by flash chromatography (FC) using neutral Al_2O_3 gel (Panreac) or SiO_2 gel (Scharlau, Kieselgel 60, 0.04-0.06 mm). NMR spectra were recorded on a Bruker DPX-300 spectrometer at 298 K using partially deuterated solvents as internal standards. Multiplicities are denoted as follows: s = singlet, d = doublet, t = triplet, m = multiplet, br = broad, dd = double doublet. IR spectra were determined on a Bruker Tensor 27 (ATR device) spectrometer. Only neat picks are reported. UV/Vis spectra were recorded with a Shimadzu Spectrophotometer UV-3600. MALDI-TOF experiments were taken on a Brucker Ultraflex III using DCTB + NaI as matrix. Femtosecond transient absorption studies were performed with 150 fs laser pulses (1 kHz) from amplified Ti:Sapphire laser systems (CPA-2101 and CPA-2110 from Clark-MXR, Inc.), the laser energy was 200 nJ.

2. General Synthetic Scheme

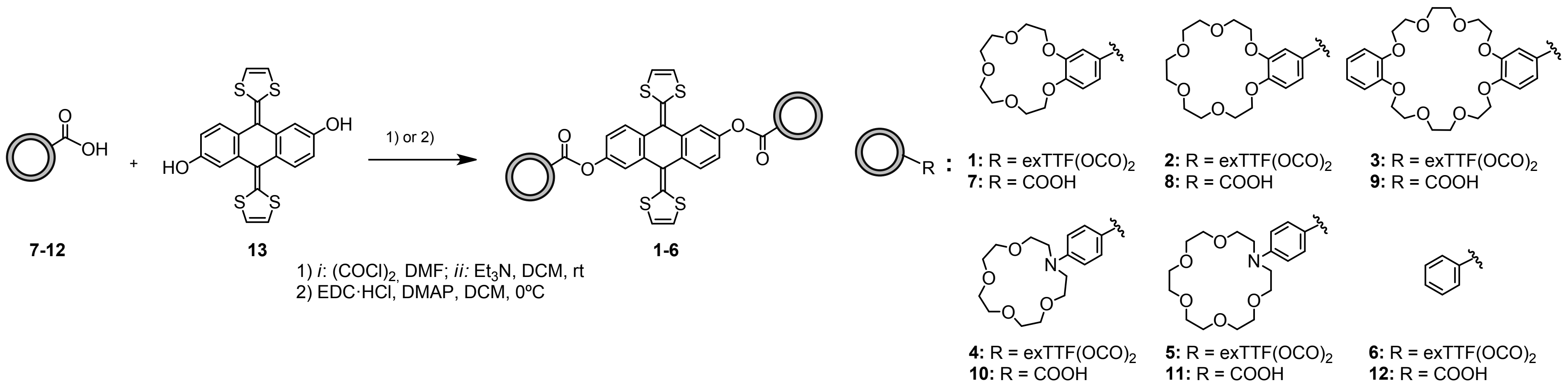

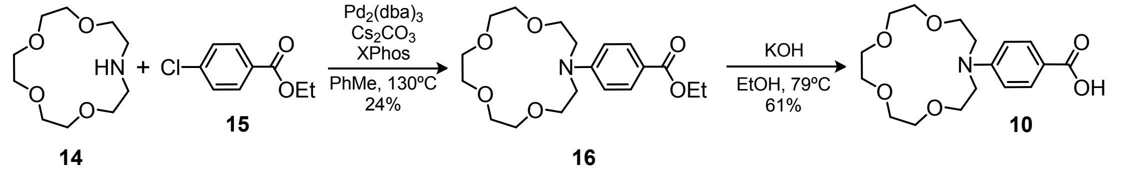

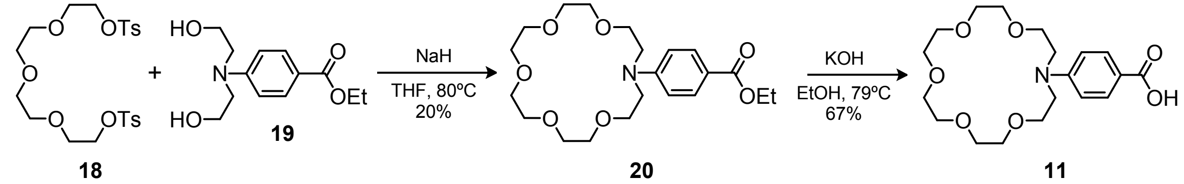

3. Synthesis

Ethyl *N-(*phenylaza[15]crown-5)-4'-carboxylate, 16.

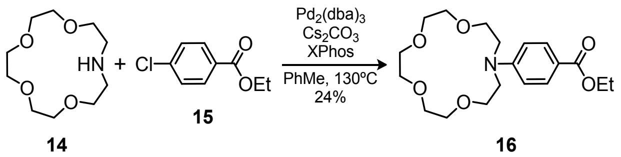

A solution of ethyl 4-chlorobenzoate **15** (0.21 mL, 1.37 mmol), aza[15]crown-5 **14** (300 mg, 1.37 mmol), $Pd_2(dba)_3$ (37 mg, 0.04 mmol), Cs_2CO_3 (500 mg, 1.53 mmol) and XPhos (78 mg, 0.164 mmol) in dry toluene (10 mL) was deoxygenated via three freeze-pump-thaw cycles, then heated in a MW at 130 °C for 1 h. The resulting mixture was filtered (Celite, DCM) and concentrated *in vacuo*. The resulting residue was taken in DCM and washed with H_2O. The organic phase was dried (Na_2SO_4), filtered, and concentrated under reduced pressure. FC (Al_2O_3, DCM/hexane, 2:1 then DCM/EtOH, 50:1) gave the desired crown ether **16** (159 mg, 24%) as a white solid. ^1H NMR (300 MHz, $CDCl_3$) δ: 7.89 (d, J = 9.1 Hz, 2H), 6.67 (d, J = 9.1 Hz, 2H), 4.31 (q, J = 7.1 Hz, 2H), 3.85–3.55 (m, 20H), 1.35 (t, J = 7.1 Hz, 3H). ^{13}C NMR (75 MHz, $CDCl_3$) δ: 167.1, 151.2, 131.5, 117.5, 110.7, 77.6, 77.2, 76.7, 71.5, 70.5, 70.3, 68.5, 60.2, 52.8, 14.6. MS (MALDI) *m/z*: 390.1 $[M + Na]^+$. HRMS (ESI): *m/z* calcd for $C_{19}H_{30}NO_6$ 368.2067. Found 368.2082.

N-(phenylaza[15]crown-5)-4'-carboxylic acid, 10.[7]

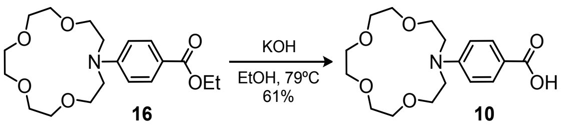

To a solution of crown ether **16** (272 mg, 0.74 mmol) in 10 mL of EtOH/H_2O (9:1), KOH (250 mg, 4.44 mmol) was added. The reaction mixture was heated under reflux for 1 h. Upon completion of the reaction, the solvent was removed to give an off-white solid, which was redissolved in H_2O (20 mL) and neutralized with aq HCl 1 M. The solution was extracted with AcOEt, and the organic layers were combined, dried (Na_2SO_4), filtered, and concentrated *in vacuum* to give **10** as a white solid of enough purity to continue with the next step (154 mg, 61 %). ^1H NMR (300 MHz, $CDCl_3$) δ: 7.93 (d, J = 9.0 Hz, 2H), 6.64 (d, J = 9.1 Hz, 2H), 3.78 (m, 4H), 3.65 (m, 16H). ^{13}C NMR (75 MHz, $CDCl_3$) δ: 172.00, 151.83, 132.30, 116.15, 110.72, 77.58, 77.16, 76.74, 71.46, 70.42, 70.22, 68.38, 52.89, 29.83, 14.23. MS (MALDI) *m/z*: 362.2 $[M + Na]^+$. HRMS (MALDI): *m/z* calcd for $C_{17}H_{25}NNaO_6$ 362.1574. Found 362.1558.

Ethyl *N*-(phenylaza[18]crown-6)-4'-carboxylate, 20.

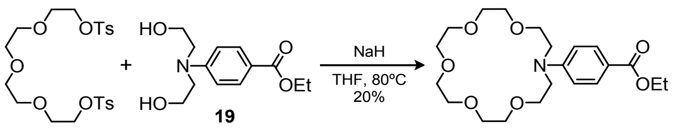

18 20

To a suspension of NaH (152 mg, 6.33 mmol) in anhydrous THF (20 mL) at 0 °C, a solution of **19** (400 mg, 1.58 mmol) in THF (10 mL) was added dropwise. The resulting mixture was heated at 30-40 °C for 1.5 h. Then a solution of tetraethyleneglycol ditosylate **18** (800 mg, .74 mmol) in THF was added and the reaction was heated at 80 °C for 5 days. After evaporating the solvent, the resulting residue was purified by FC (SiO_2, DCM/MeOH, 200:1) yielding **20** as a transparent oil (131.3 mg, 20 %). ^{1}H NMR (300 MHz, $CDCl_3$) δ: 7.80 (d, J = 9.0 Hz, 2H), 6.57 (d, J = 9.0 Hz, 2H), 4.23 (q, J = 7.1 Hz, 2H), 3.67–3.52 (m, 24H), 1.28 (t, J = 7.0 Hz, 3H). ^{13}C NMR (75 MHz, $CDCl_3$) δ: 166.9, 151.3, 131.4, 117.3, 110.5, 70.9, 70.8, 70.8, 70.7, 70.6, 69.8, 68.5, 66.6, 60.1, 51.3, 14.5. MS (ESI) *m/z*: 434.2 $[M + Na]^+$. HRMS (ESI): *m/z* calcd for $C_{21}H_{34}NO_7$ 412.2329. Found 412.2329.

***N*-(Phenylaza[18]crown-6)-4'-carboxylic acid, 11.**

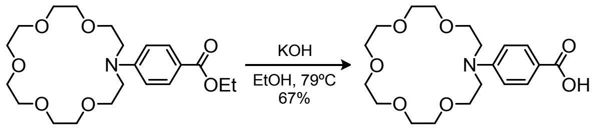

20 11

A 100 mL one-necked, round-bottomed flask was charged with ethyl *N*-(phenylaza[18]crown-6)-4'-carboxylate **20** (130 mg, 0.32 mmol) and EtOH (30 mL). A solution of aq KOH (107 mg, 1.91 mmol in 10 mL) was added dropwise and the reaction mixture was heated at reflux for 12 h. Upon completion of the reaction the solvent was removed to give an off-white solid, which was redissolved in H_2O (50 mL) and neutralized with H_2SO_4. The solution was extracted with DCM, and the organic layers were combined, dried over Na_2SO_4, and concentrated to give **11** as a white solid, which was employed in the next step without further purification (82 mg, 67 %). ^{1}H NMR (300 MHz, $CDCl_3$) δ: 7.94 (d, J = 8.94 Hz, 2H), 6.67 (d, J = 8.94 Hz, 2H), 3.72–3.67 (m, 24H). ^{13}C NMR (75 MHz, $CDCl_3$) δ: 171.9, 152.0, 132.3, 116.1, 110.7, 71.0, 70.9, 70.9, 70.9, 70.8, 70.7, 69.9, 68.5, 66.7, 51.4. MS (ESI) *m/z*: 381.9 $[M - H]^-$. HRMS (ESI): *m/z* calcd for $C_{19}H_{30}NO_6$ 368.2067. Found 368.2082.

2,6-*Bis*[(benzo[15]crown-5)-4'-carbonyloxy]exTTF, 1.

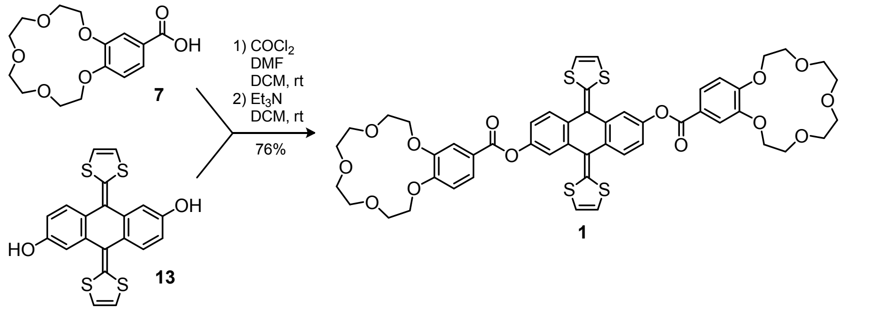

To a stirring solution of (benzo[15]crown-5)-4'-carboxylic acid **8** (100 mg, 0.32 mmol) and a catalytic amount of DMF in anhydrous DCM (20 mL), oxalyl chloride (0.1 mL, 0.93 mmol) was added at rt. After 30 min, the solvent was removed under reduced pressure, the resulting residue was diluted with anhydrous DCM (30 mL) and Et_3N (0.1 mL, 0.66 mmol) and 2,6-dihydroxy-exTTF **13** (62 mg, 0.15 mmol) were added. The reaction mixture was stirred at rt until no precipitate was observed. Purification of the reaction crude was performed by FC (Al_2O_3, DCM/MeOH, 100:0.2). The resulting product **2** was obtained as a yellow powder (38 mg, 76%). ^{1}H NMR (300 MHz, $CDCl_3$) δ: 7.43 (dd, J = 8.4, 1.90 Hz, 2H), 7.72 (d, J = 8.5 Hz, 2H), 7.69 (d, J = 1.9 Hz, 2H), 7.57 (d, J = 2.3 Hz, 2H), 7.12 (dd, J = 8.4, 2.34 Hz, 2H), 6.93 (d, J = 8.5 Hz, 2H), 6.31 (s, 4H), 4.25-4.22 (m, 8H), 3.97-3.92 (m, 8H), 3.79–3.78 (m, 16H). ^{13}C NMR (75 MHz, $CDCl_3$) δ: 165.1, 154.1, 159.1, 148.9, 137.2, 137.1, 133.3, 126.1, 125.1, 122.4, 121.0, 119.2, 118.7, 117.7, 117.5, 115.3, 112.4, 71.5, 70.7, 70.6, 69.7, 69.4, 69.0. FTIR (DCM) ν: 2870, 1728, 1599, 1547, 1512, 1463, 1428, 1347, 1270, 1188, 1135, 1059, 960, 931, 755, 656 cm^{-1} UV-vis (DCM) λ_{max} (log ε): 353 (4.04), 368 (4.22), 420 (4.36), 436 (4.42) nm. MS (MALDI) m/z: 1023.2 [M + Na]$^+$, 1000.2 [M]$^+$. HRMS (MALDI): m/z calcd for $C_{50}H_{48}NaO_{14}S_4$ 1023.1819. Found 1023.1810. TGA: weight loss (temperature desorption/decomposition): 39.7% (345ºC), 23.2% (384ºC).

2,6-*Bis*[(dibenzo[24]crown-8)-4'-carbonyloxy]exTTF, 3.

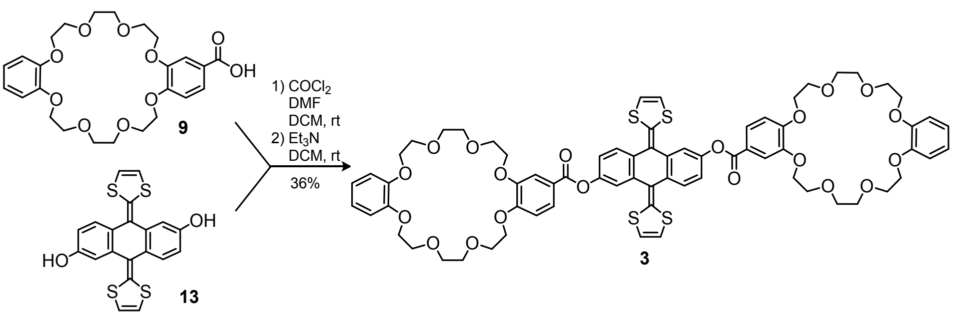

To a stirring solution of carboxylic acid **9** (125 mg, 0,25 mmol) and a catalytic amount of DMF in anhydrous DCM (20 mL), oxalyl chloride (0.1 mL, 0.93 mmol) was added. After 30 min at rt, the solvent was removed under reduced pressure, the resulting residue was diluted with anhydrous DCM (30 mL) and Et_3N (0.1 mL, 0.66 mmol) and 2,6-dihydroxy-exTTF **13** (50 mg, 0.12 mmol) were added.

The reaction mixture was stirred at rt until no precipitate was observed. Purification of the reaction crude was performed by subsequent FC (SiO_2, DCM/MeOH/NH_3, 90:10:0.3) and FC (Al_2O_3, DCM/MeOH, 200:1), obtaining the titled product **3** (60 mg, 36%) as a yellow powder. ^{1}H NMR (300 MHz, $CDCl_3$) *δ*: 7.86 (dd, *J* = 8.4, 2.0 Hz, 2H), 7.72 (d, *J* = 8.4 Hz, 2H), 7.69 (d, *J* = 2.0 Hz, 2H), 7.58 (d, *J* = 2.3 Hz, 2H), 7.12 (dd, *J* = 8.4, 2.3 Hz, 2H), 6.93 (d, *J* = 8.4 Hz, 2H), 6.89 (m, 8H), 6.31 (s, 4H), 4.25–4.22 (m, 8H), 4.17–4.15 (m, 8H), 3.98–3.91 (m, 16H), 3.86–3.84 (m, 16H). ^{13}C NMR (75 MHz, $CDCl_3$) *δ*: 164.9, 153.9, 148.9, 148.7, 137.1, 136.9, 133.1, 126.0, 125.0, 122.2, 120.8, 119.0, 118.5, 117.6, 117.3, 115.1, 112.3, 71.3, 70.6, 70.5, 69.5, 69.4, 69.2, 68.8. FTIR (DCM) *v*: 2924, 2856, 1726, 1596, 1505, 1454, 1428, 1264, 1187, 1128, 1054, 960, 733, 701 cm^{-1}. UV-vis (DCM) λ_{max} (log ε): 353 (3.98), 369 (4.14), 421 (4.25), 437 (4.31) nm. MS (MALDI) *m/z*: 1383.3 [M + Na]$^+$. HRMS (MALDI): *m/z* calcd for $C_{70}H_{72}NaO_{20}S_4$: 1383.3392. Found: 1383.3371. TGA: weight loss (temperature desorption/decomposition): 16.1% (107-302ºC), 20.2% (368ºC), 41.9% (410ºC).

2,6-*Bis*[(*N*-phenylaza[15]crown-5)-4'-carbonyloxy]exTTF, 4.

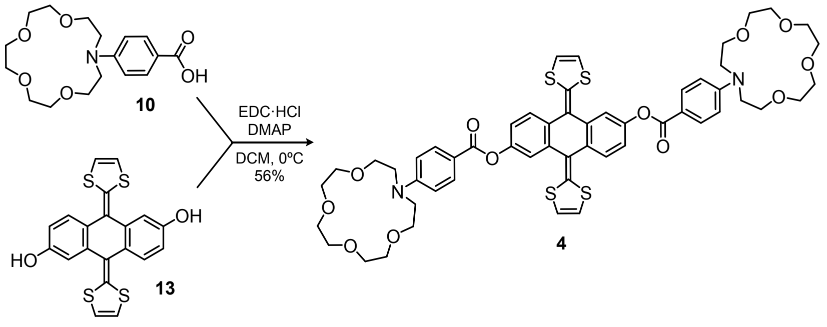

To a stirring solution of 1,6-dihydroxy-exTTF **13** (40 mg, 0.10 mmol) and carboxylic acid **10** (70 mg, 0.21 mmol) in dry DCM (20 mL) at 0 °C, EDC·HCl (57 mg, 0.30 mmol) and DMAP (37 mg, 0.30 mmol) were added portionwise. The resulting solution was allowed to warm up to rt and then stirred overnight. The reaction mixture was diluted with 20 mL of DCM and sequentially washed with $NaHCO_3$ sat. aq solution, aq HCl 1 M, and H_2O, dried over Na_2SO_4, filtered, and evaporated under reduced pressure. The resulting residue was purified by FC (Al_2O_3, DCM/MeOH, 100:0.2). Receptor **4** was obtained as a yellow solid (58 mg, 56 %). ^{1}H NMR (300 MHz, $CDCl_3$) *δ*: 8.05 (d, *J* = 8.6 Hz, 4H), 7.69 (d, *J* = 8.4 Hz, 2H), 7.56 (d, *J* = 2.4 Hz, 2H), 7.11 (dd, *J* = 8.4, 2.4 Hz, 2H), 6.70 (d, *J* = 8.6 Hz, 4H), 6.30 (s, 4H), 3.80–3.78 (m, 8H), 3.70–3.64 (m, 32H). ^{13}C NMR (75 MHz, $CDCl_3$) *δ*: 165.35, 149.23, 136.87, 136.53, 132.82, 132.38, 125.88, 121.16, 119.20, 118.64, 117.62, 117.24, 116.19, 110.85, 71.49, 70.47, 70.26, 68.35, 52.95. FTIR (DCM) *v*: 2865, 1716, 1601, 1547, 1521, 1465, 1394, 1353, 1263, 1176, 1124, 1062, 995, 829, 761, 732, 699, 659 cm^{-1}. UV-vis (DCM) λ_{max} (log *ε*): 324 (4.90), 368 (4.25), 417 (4.33), 435 (4.40) nm. MS (MALDI) *m/z*: 1077.3 [M + Na]$^+$, 1054.3 [M]$^+$. HRMS (MALDI) *m/z* calcd for $C_{54}H_{58}N_2O_{12}S_4$ 1054.2867. Found 1054.2873. TGA: weight loss (temperature

desorption/decomposition): 43.7% (147ºC), 23.7% (341ºC).

2,6-*Bis*[(*N*-phenylaza[18]crown-6)-4'-carbonyloxy]exTTF, 5.

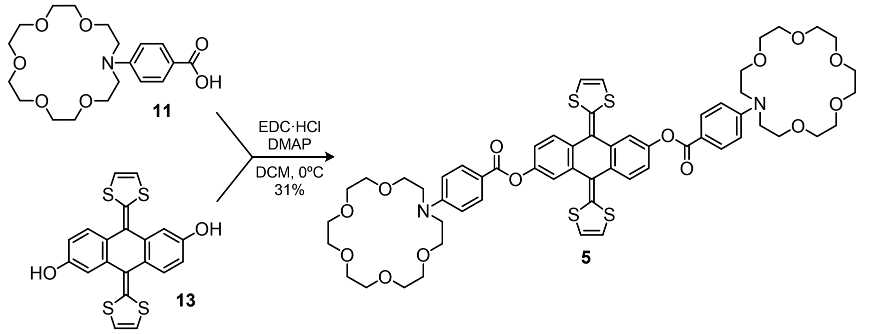

To a stirring solution of 1,6-dihydroxy-exTTF **13** (41 mg, 0.10 mmol) and carboxylic acid **11** (81 mg, 0,21 mmol) in dry DCM (20 mL) at 0 °C, EDC hydrochloride (42 mg, 0.22 mmol) and DMAP (27 mg, 0.22 mmol) were added portionwise. The resulting solution was allowed to slowly warm up to rt and then stirred overnight. The reaction mixture was diluted with 20 mL of DCM and sequentially washed with Na_2CO_3 sat. aq solution, HCl 1 M, and $NaHCO_3$ sat. solution, dried over Na_2SO_4, filtered, and evaporated under reduced pressure. The resulting residue was purified by FC (Al_2O_3, DCM/MeOH, 100:0.2). Receptor **5** was obtained as a yellow solid (33 mg, 31 %). ^{1}H NMR (300 MHz, $CDCl_3$) δ: 8.04 (d, J = 9.0 Hz, 4H), 7.69 (d, J = 8.4 Hz, 2H), 7.56 (d, J = 2.3 Hz, 2H), 7.10 (dd, J = 8.4, 2.3 Hz, 2H), 6.72 (d, J = 9.0 Hz, 4H), 6.29 (d, J = 1.0 Hz, 4H), 3.74–3.73 (m, 16H), 3.67–3.66 (m, 32H). ^{13}C NMR (75 MHz, $CDCl_3$) δ: 165.3, 152.0, 149.2, 136.8, 136.5, 132.8, 132.4, 125.8, 121.1, 119.2, 118.6, 117.6, 117.2, 116.1, 110.8, 77.6, 77.2, 76.7, 71.0, 70.9, 70.9, 70.9, 68.5, 51.5. FTIR (DCM) v: 2919, 2860, 1716, 1602, 1522, 1464, 1403, 1351, 1263, 1176, 1110, 1062, 995, 760, 732, 699 cm^{-1}. UV-vis (DCM) λ_{max} (log ε): 325 (4.96), 368 (4.32), 418 (4.40), 435 (4.46) nm. MS (MALDI) m/z: 1165.3 [M + Na]$^+$, 1142.3 [M]$^+$. HRMS (MALDI) m/z calcd for $C_{58}H_{66}N_2NaO_{14}S_4$ 1165.3289. Found 1165.3290. TGA: weight loss (temperature desorption/decomposition): 36.0% (338ºC), 28.9% (375ºC).

2,6-*Bis*[benzoate-4'-carbonyloxy]exTTF, 6.

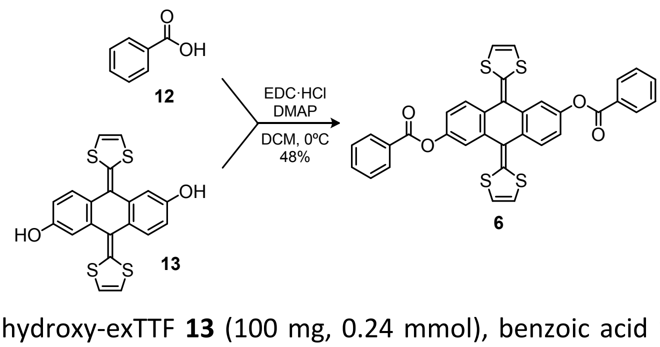

To a solution of 2,6-dihydroxy-exTTF **13** (100 mg, 0.24 mmol), benzoic acid **12** (62 mg, 0.51 mmol) and DMAP (74 mg, 0.61 mmol) in dried DCM (50 mL), EDC hydrochloride (116 mg, 0.61 mmol) was

added portionwise at 0 °C. The resulting mixture was stirred for 2 h at rt. After evaporation of the solvent under reduced pressure, the mixture was purified by FC (SiO_2, DCM/MeOH, 100:4). Compound **6** was obtained as a yellow solid (71 mg, 48%). ^1H NMR (300 MHz, $CDCl_3$) δ: 8.29–8.20 (m, 4H), 7.74 (d, J = 8.4 Hz, 2H), 7.70–7.61 (m, 2H), 7.60 (d, J = 2.4 Hz, 2H), 7.58–7.48 (m, 4H), 7.15 (dd, J = 8.4, 2.4 Hz, 2H), 6.32 (s, 4H). ^{13}C NMR (75 MHz, $CDCl_3$) δ: 165.2, 148.8, 137.2, 137.0, 133.8, 133.2, 130.4, 129.7, 128.7, 126.0, 120.7, 119.0, 118.4, 117.6, 117.3, 77.6, 77.4, 77.2, 76.7. FTIR (DCM) v: 2922, 1735, 1547, 1509, 1465, 1258, 1189, 1025, 799, 708 cm^{-1}. UV-vis (DCM) λ_{max} (log ε): 353 (4.22), 369 (4.40), 420 (4.53), 437 (4.60) nm. MS (MALDI) m/z: 620.1 $[M]^+$. HRMS (MALDI): m/z calcd for $C_{34}H_{20}O_4S_4$ 620.0239. Found 620.0220. TGA: weight loss (temperature desorption/decomposition): 20.5% (215ºC), 23.8% (338ºC) , 22.7% (383-660 ºC).

4. Titration Experiments

UV-visible titrations experiments were performed by adding increasing quantities of C_{60} to a solution of the corresponding exTTF-(crown ether)$_2$ receptor in PhCl. Absorption measurements were realized following two different approaches:

- First, to a solution of exTTF-(crownether)$_2$ in a 1x1cm quartz cuvette a solution containing the same concentration of exTTF-(crownether)$_2$ and a higher concentration of C_{60} was added stepwise (Figure S1a). With this, it was assured that the exTTF-(crownether)$_2$ concentration was held constant throughout the whole titration while increasing the fullerene concentration. At each step, UV-vis absorption spectra were acquired. Analysis of the experimental data was performed monitoring the spectroscopic changes of the exTTF-centered absorption band and the corresponding evolving charge transfer bands performing a non-linear curve fitting with OriginLab Origin 9.0 software.
- In the second approach (Figure S1b), for each of the titration point a new solution was prepared in amber vials. Stock solutions of host and guest molecules were degassed by bubbling Ar through for several minutes before the measurement. Best reproducibility was obtained by adding C_{60} immediately prior to the measurement. In order to ensure that most of the measurements were made in the 20-80% complexation range, the p-value was calculated for each titration point ensuring that they remained in the 0.2–0.8 range. The widest range of p-values was obtained by working with a host concentration of approximately one-tenth of the dissociation constant and added a large excess of guest molecule.[8] The addition of large C_{60} excess hampered the observation of the spectroscopic induced complexation changes. Analysis of the experimental data was made by non-linear curve fitting software SPECFIT/32™.[9]

Values obtained by both approaches laid in the same range so the first, simpler, method was used in the rest of experiments. Binding constants in Table 1 (main text) arise from calculating the average value of the experimental results, their error (σ), is defined as the standard deviation.

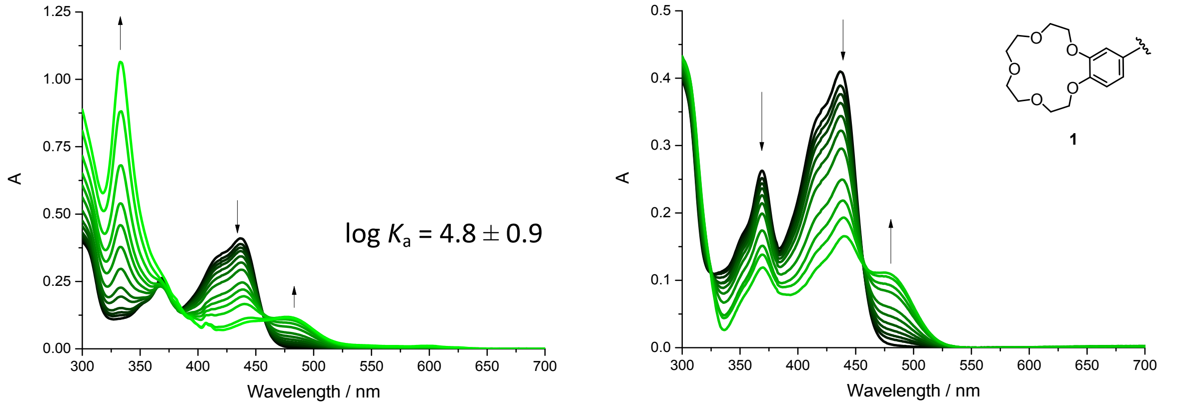

[**1**] = 1.5×10^{-5} M. [C_{60}] = 0, 2.5x10^{-7}, 5.0x10^{-7}, 9.9x10^{-7}, 2.0x10^{-6}, 2.9x10^{-6}, 4.8x10^{-6}, 7.0x10^{-6}, 9.1x10^{-6}, 1.1x10^{-5}, 1.5x10^{-5}, 2.0x10^{-5}, 2.5x10^{-5} M

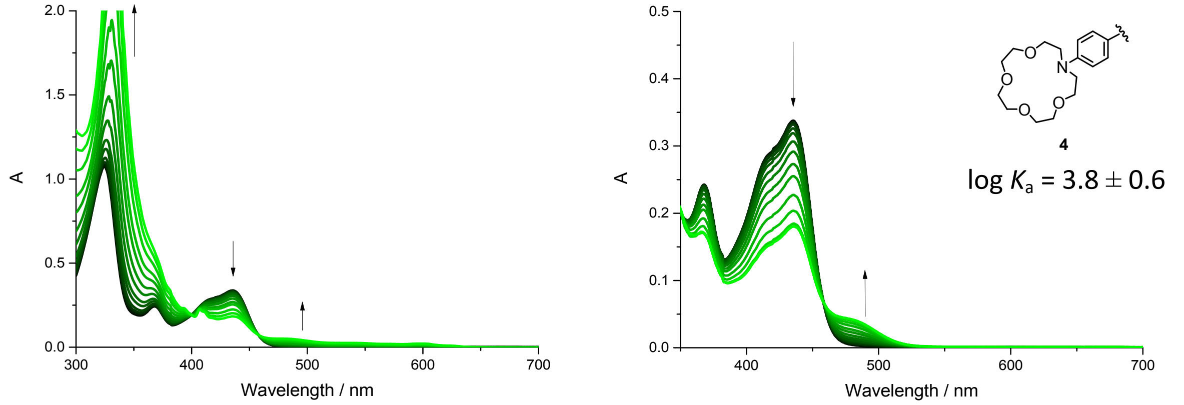

[**4**] = 1.5×10^{-5} M. [C_{60}] = 0, 3.5x10^{-7}, 6.9x10^{-7}, 1.4x10^{-6}, 2.7x10^{-6}, 4.0x10^{-6}, 6.4x10^{-6}, 9.1x10^{-6}, 1.3x10^{-5}, 1.6x10^{-5}, 2.1x10^{-5}, 2.6x10^{-5}, 3.0x10^{-5} M

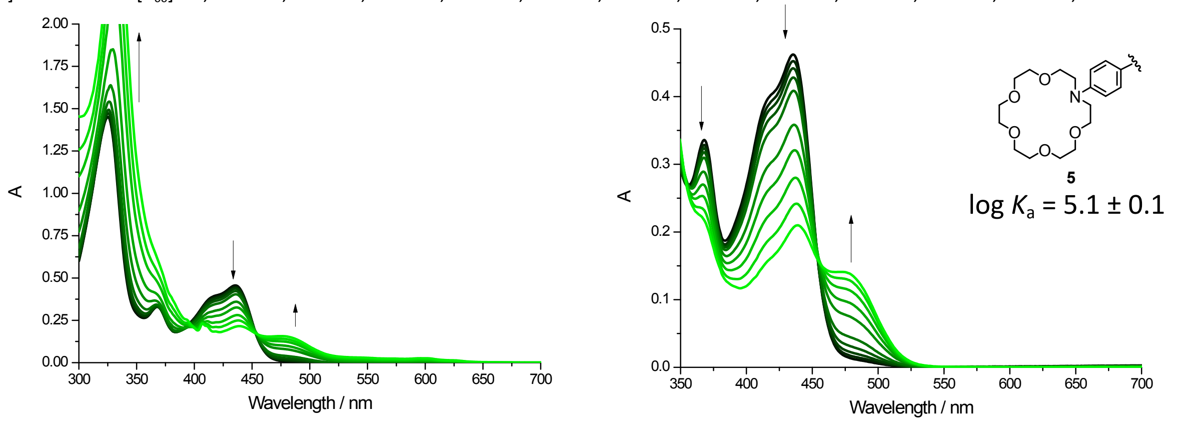

[**5**] = 1.5×10^{-5} M. [C_{60}] = 0, 3.5x10^{-7}, 6.9x10^{-7}, 1.4x10^{-6}, 2.7x10^{-6}, 5.2x10^{-6}, 9.7x10^{-6}, 1.7x10^{-5}, 2.3x10^{-5}, 3.0x10^{-5} M

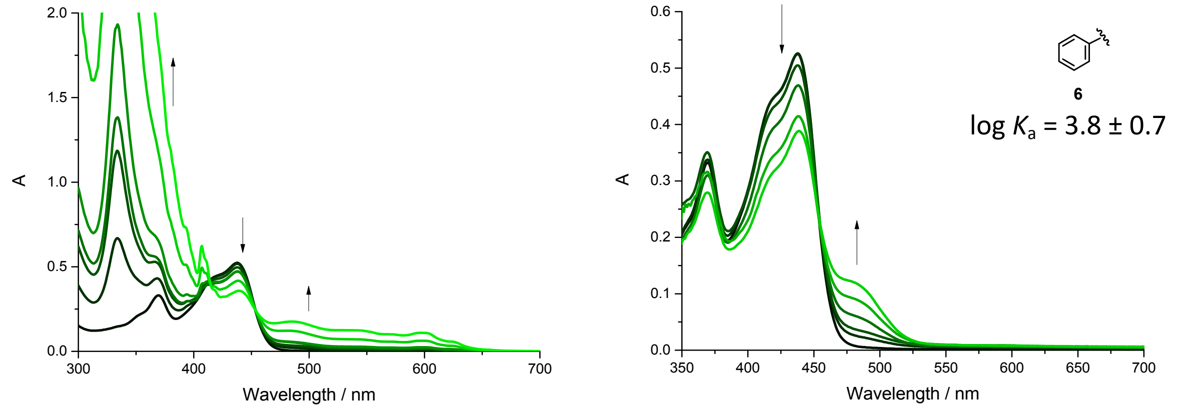

[**6**] = 1.5×10^{-5} M. [C_{60}] = 0, 6.9x10^{-6}, 1.2x10^{-5}, 1.7x10^{-5}, 2.5x10^{-5}, 3.3x10^{-5}, 6.4x10^{-5} M

Figure S1a. *Left part* – Raw UV-vis absorption spectra of **1**-**6** upon addition of increasing quantities of C_{60} in PhCl at rt. *Right part* – Spectra upon subtraction of C_{60} absorption.

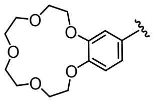

[**1**] = 1.14×10^{-5} M.

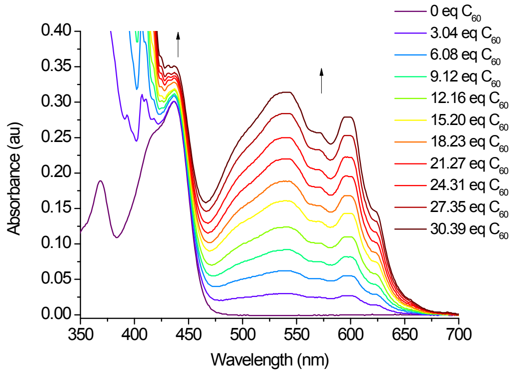

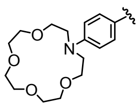

[**4**] = 1.02×10^{-4} M.

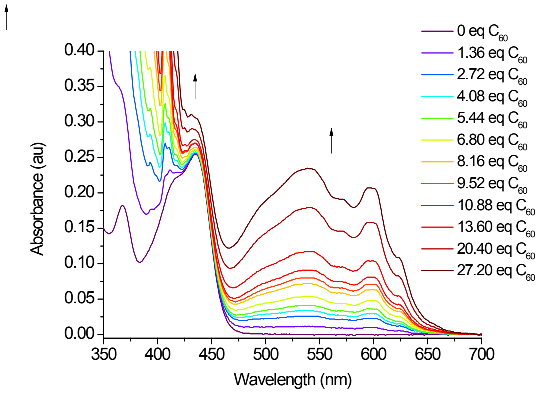

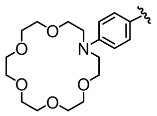

[**5**] = 1.02×10^{-4} M.

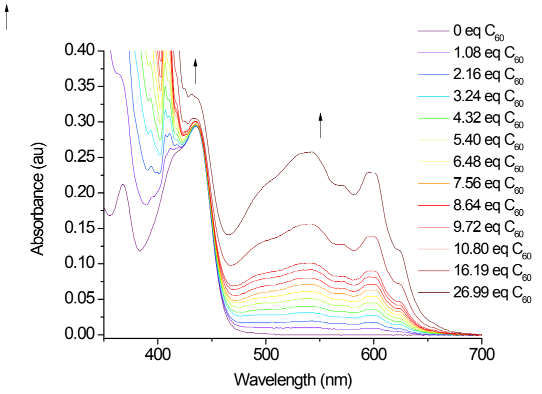

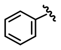

[**6**] = 1.07×10^{-4} M.

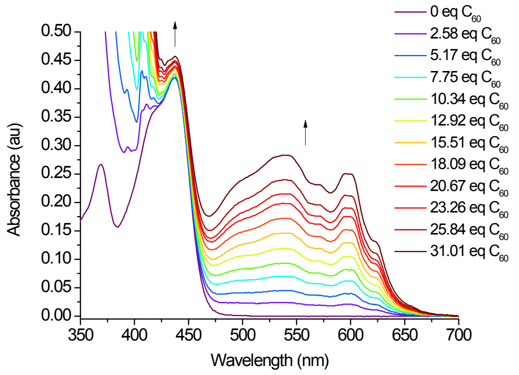

Figure S1b. UV-vis spectral changes observed during the complexation of **1-6** by addition of C_{60} in PhCl at rt. Arrows indicate the progression of the titration.

5. Complementary spectroscopical analysis

UV-vis absorption experiments in PhCN

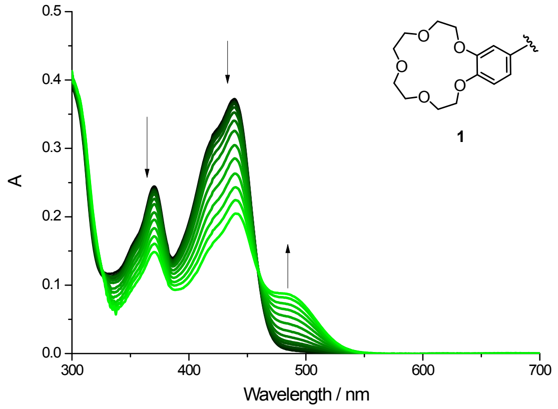

[**1**] = 1.5×10^{-5} M. [C_{60}] = 0 up to 3.0x10^{-5} M

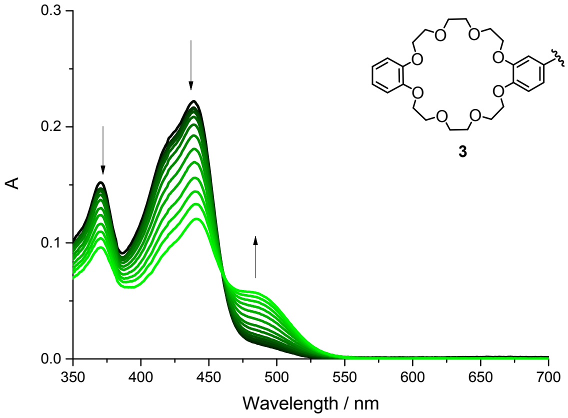

[**3**] = 1.5×10^{-5} M. [C_{60}] = 0 to 3.0x10^{-5} M

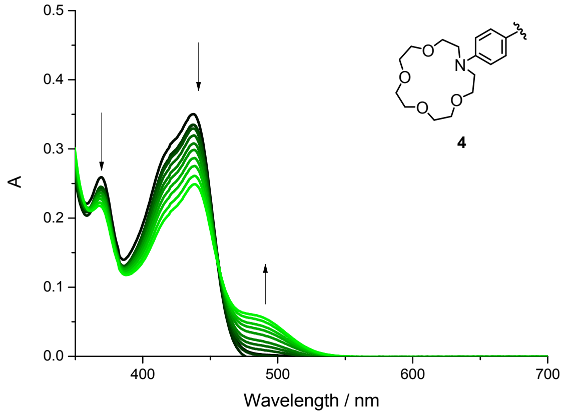

[**4**] = 1.5×10^{-5} M. [C_{60}] = 0 to 1.0 x 10^{-5} M

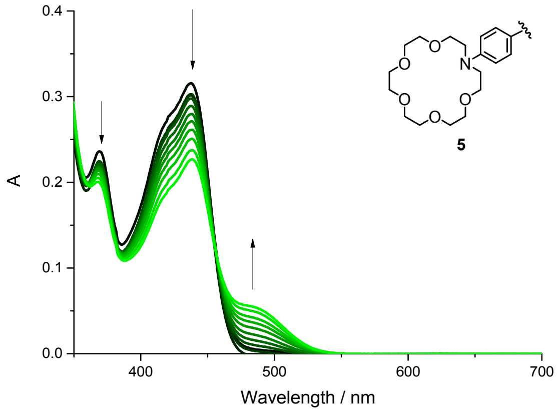

[**5**] = 1.5×10^{-5} M. [C_{60}] = 0 to 1.0 x 10^{-5} M

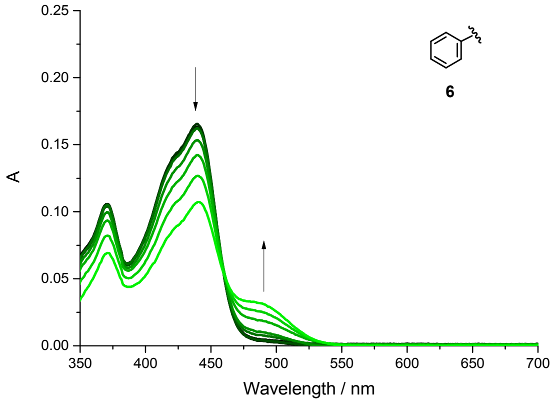

[**6**] = 1.0×10^{-5} M. [C_{60}] = 0 to 1.0 x 10^{-5} M

Figure S2. UV-vis spectroscopical changes of **1-6** upon increasing addition of C_{60}. Fullerene absorption has been subtracted. Arrows indicate the progression of the observed changes.

Emission experiments in PhCl and PhCN

PhCl

PhCN

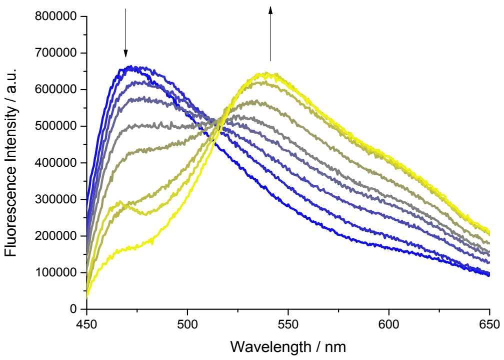

λ_{exc} = 350 nm. [**1**] = 1.5×10^{-5} M. [C_{60}] = 0 to 7.0 x 10^{-6} M

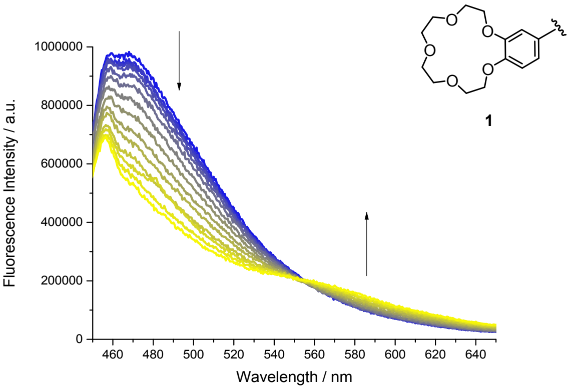

λ_{exc} = 400 nm. [**1**] = 1.5×10^{-5} M. [C_{60}] = 0 to 3.0 x 10^{-5} M

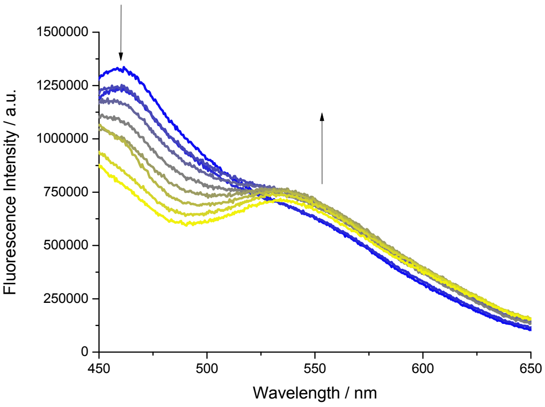

λ_{exc} = 350 nm. [**3**] = 1.5×10^{-5} M. [C_{60}] = 0 to 7.0 x 10^{-6} M

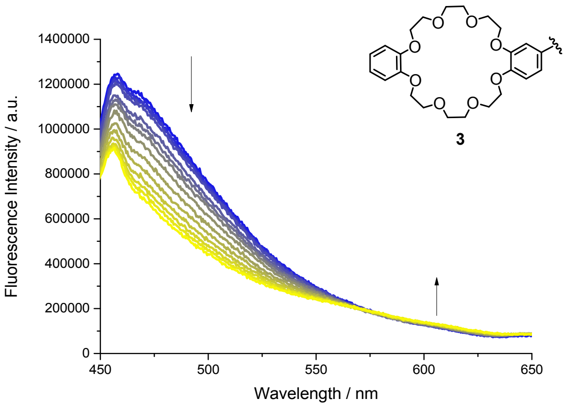

λ_{exc} = 450 nm. [**1**] = 1.4 ×10^{-5} M. [C_{60}] = 0 to 2.6 x 10^{-5} M

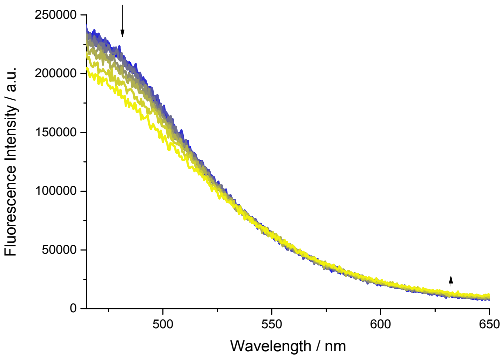

λ_{exc} = 400 nm. [**4**] = 1.5×10^{-5} M. [C_{60}] = 0 to 9.1 x 10^{-6} M

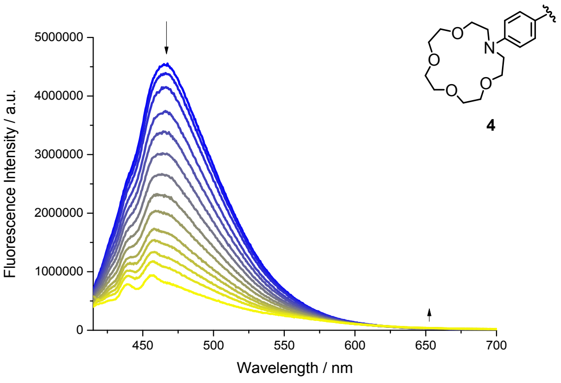

λ_{exc} = 400 nm. [**4**] = 1.5×10^{-5} M. [C_{60}] = 0 to 1.4 x 10^{-5} M

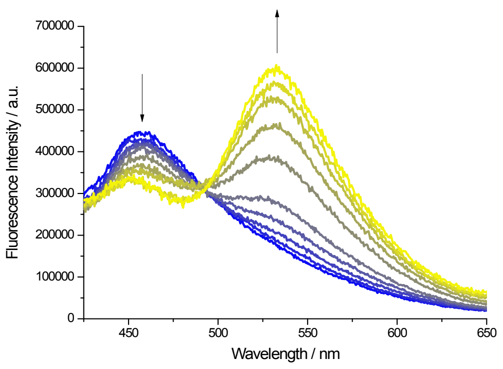

λ_{exc} = 350 nm. [**5**] = 1.5×10^{-5} M. [C_{60}] = 0 to 2.3 x 10^{-5} M

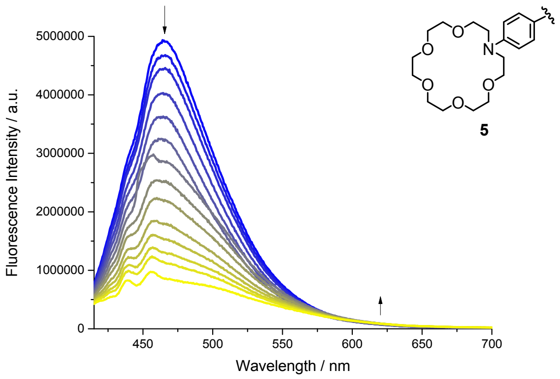

λ_{exc} = 400 nm. [**5**] = 1.5×10^{-5} M. [C_{60}] = 0 to 1.4 x 10^{-5} M

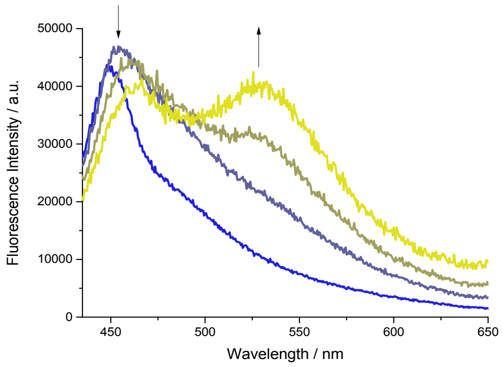

λ_{exc} = 350 nm. [**6**] = 3 × 10^{-5} M. [C_{60}] = 0 to 3.3 x 10^{-5} M

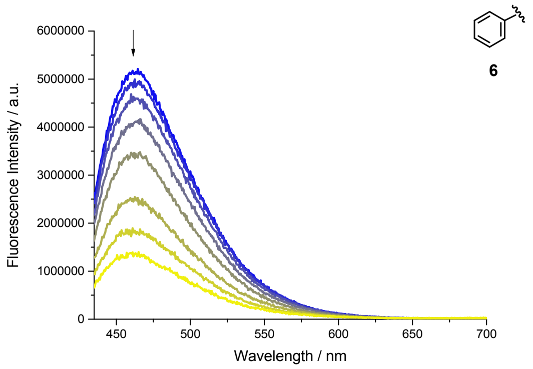

λ_{exc} = 350 nm. [**6**] = 1.5×10^{-5} M. [C_{60}] =0 to 3.0 x 10^{-5} M

Figure S3. Emission spectra of **1-6** upon increasing addition of C_{60} at rt. *Left part* –In PhCl. *Right part* – In PhCN. Arrows indicate the progression of the titration.

6. MS Spectra of Supramolecular Complexes with C_{60}.

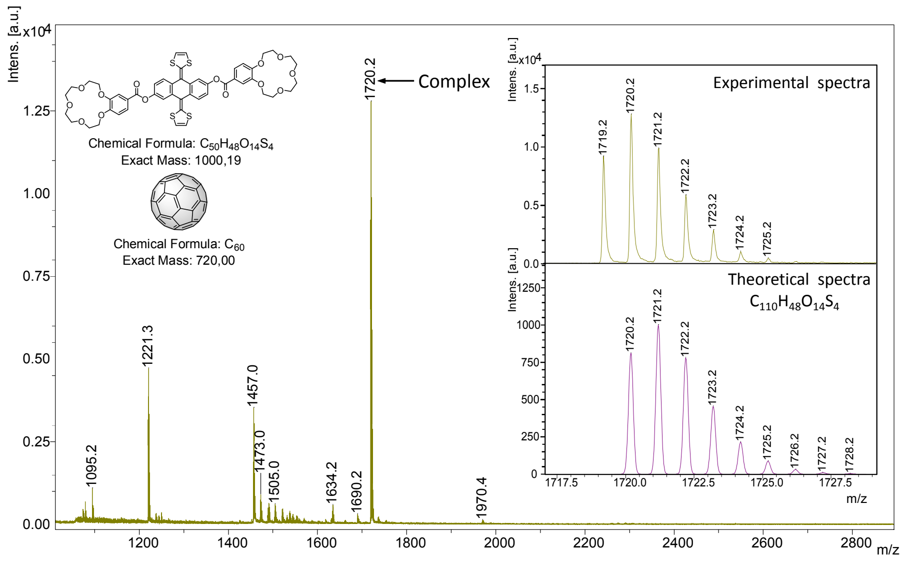

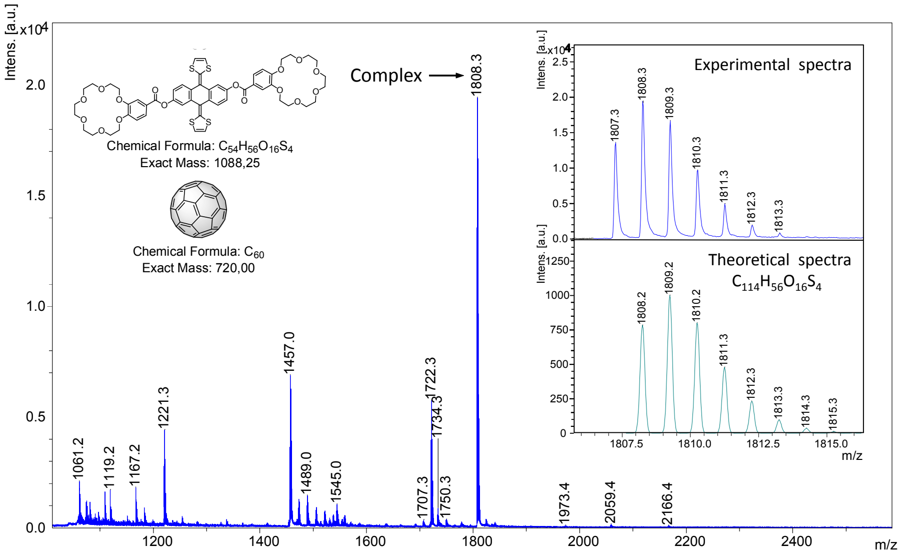

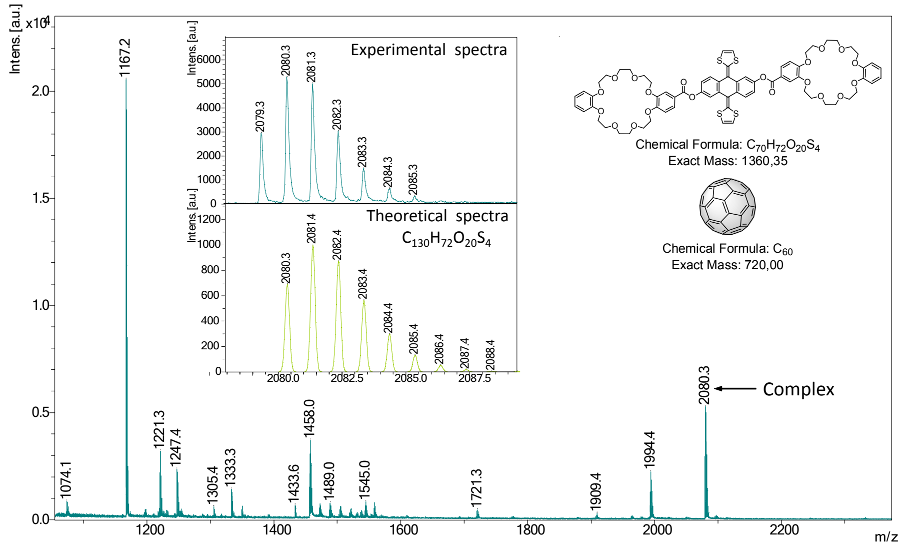

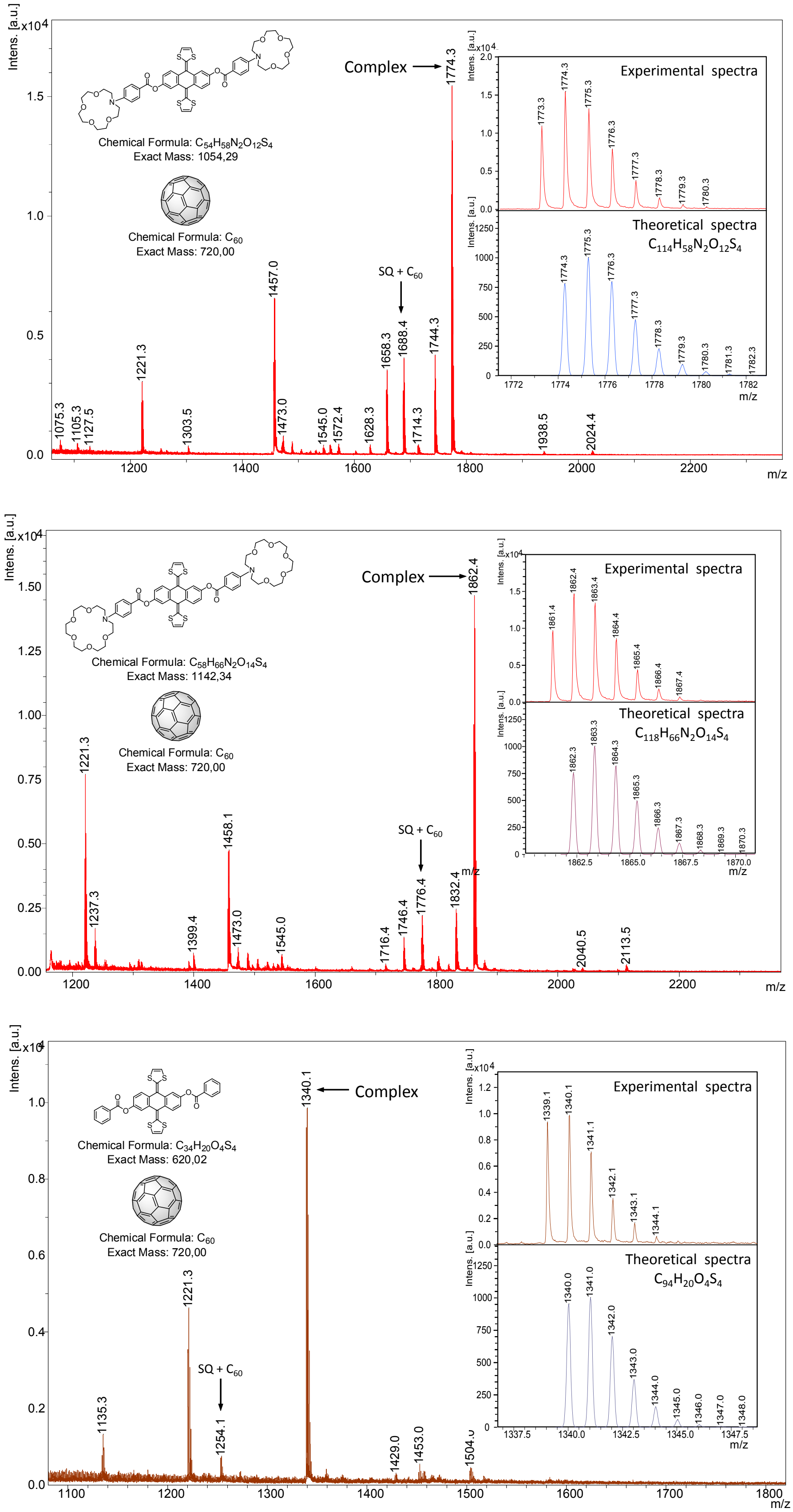

Figure S4. Mass spectra of complexes **[1–6]·C_{60}**. Spectra were obtained by mixing equimolecular amounts of the exTTF derivative **1**–**6** and C_{60} in PhCl. Solutions of the complexes were prepared immediately before their analysis. DCTB was employed as the matrix.

7. Electrochemistry

Cyclic voltammetry was made in PhCl/MeCN 4:1 at a host concentration of 5×10^{-4} M. The supporting electrolyte $[nBu_4N][BF_4]$ (0.1 M) was used as received and simply degassed under Ar. Measurements were carried out in an Autolab PGStat 30. Experiments were made in a double-walled cell (Metrohm EA 876-20).). The counter electrode was a Pt wire of ca 1 cm² apparent surface. The working electrode was a glassy carbon electrode (Metrohm 6.0804.010). The reference electrode was a $Ag/AgNO_3$ electrode. Before each measurement, the solutions were degassed with Ar and the working electrode was polished with alumina (30 µ) for 1 min.

Table S1. Redox potentials of exTTF receptors and their complexes with C_{60}. Values *vs* Ag/Ag^+ at 100 mV/s.

	E^1_{ox} [a]	E^1_{red} [b]	E^2_{red} [b]	E^3_{red} [b]	E^4_{red} [b]
**C_{60}**	—	–0.80	–1.19	–1.68	–2.16
**1**	0.22	—	—	—	—
**[1·C_{60}]**	0.37	–0.85	–1.24	–1.72	–2.23
**2**	0.26	—	—	—	—
**[2·C_{60}]**	0.33	–0.85	–1.24	–1.72	–2.22
**3**	0.23	—	—	—	—
**[3·C_{60}]**	0.36	–0.90	–1.30	–1.76	–2.28
**6**	0.20	—	—	—	—
**[6·C_{60}]**	0.30	–0.83	–1.23	–1.70	–2.19

[a] Anodic peak. [b] Cathodic peak.

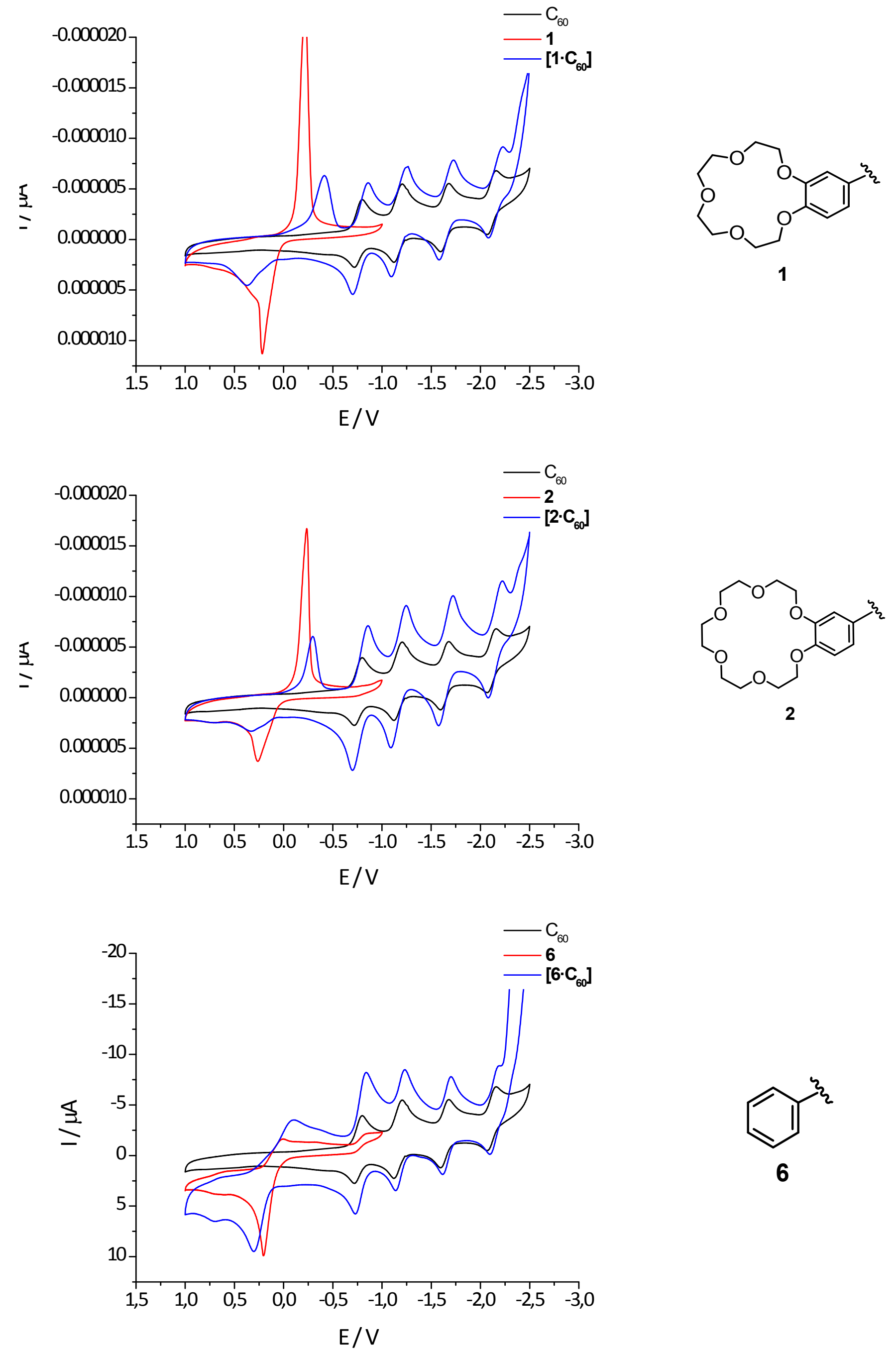

Figure S5. Cyclic voltammograms of complexes **1·C$_{60}$**, **2·C$_{60}$**, and **6·C$_{60}$**. The CVs of **1**, **2**, **6**, and **C$_{60}$** are also displayed. SR: 100 mV/s; PhCl/MeCN 4/1; SE: n-Bu$_4$NPF$_6$ (0.1 M); WE: GCE; CE: Pt wire; 298 K.

8. Transient Absorption Studies

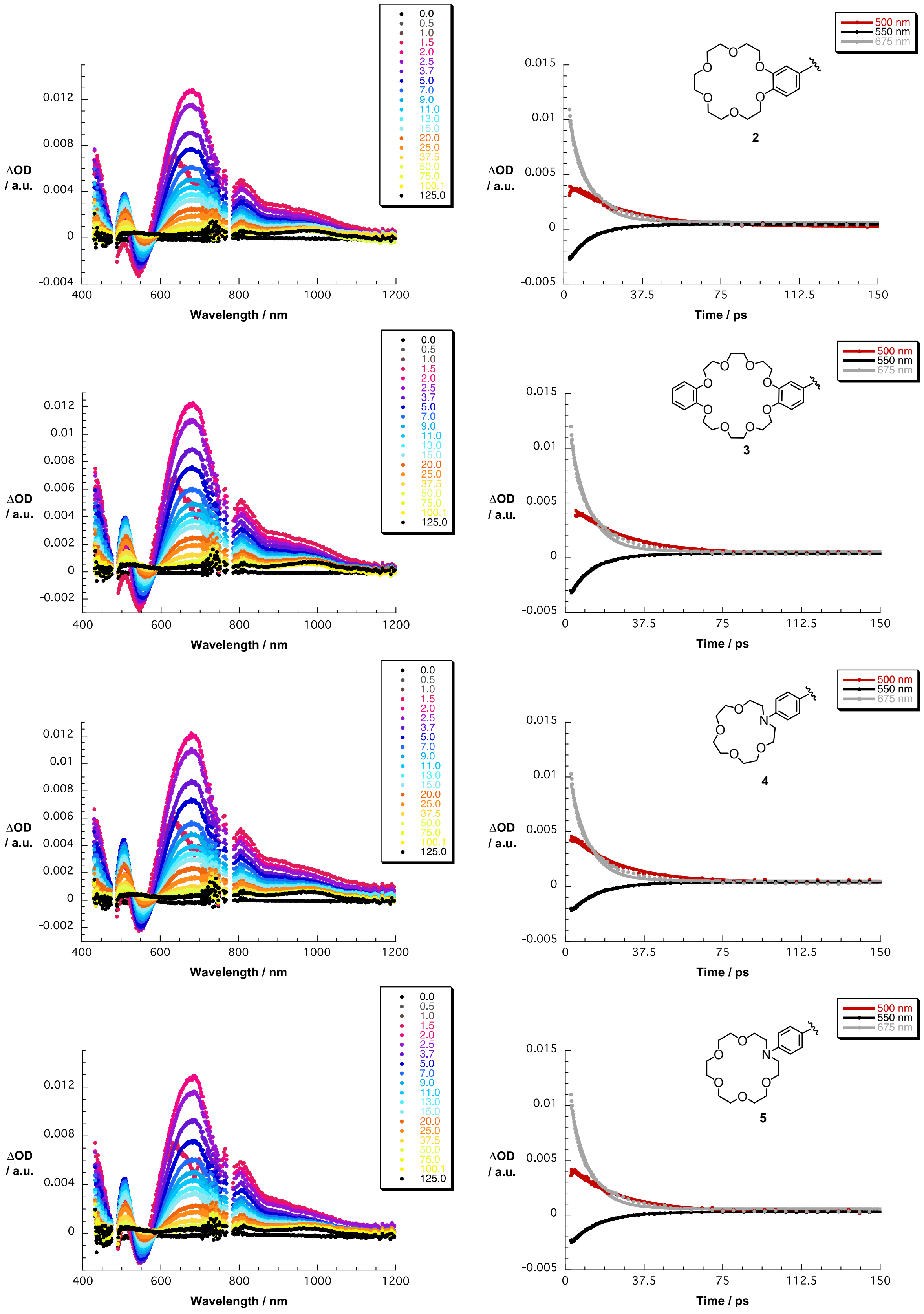

Figure S6. *Left part* – differential absorption spectrum (visible and near-infrared) obtained upon femtosecond flash photolysis (480 nm) of **2-5·C$_{60}$** (1:1) in PhCl with several time delays between 0 and 125 ps at room temperature. *Right part* – time-absorption profiles of the spectra at 500, 550, and 675 nm, monitoring the charge separation / charge recombination.

9. Theoretical Calculations

A first exploration of the supramolecular potential energy surface was carried out by performing geometry optimizations of the different **1**–**6·C$_{60}$** host-guest associates at the semiempirical PM7 level of theory[10] using the MOPAC2012 program package.[11] The geometry optimization termination criterion (gradient norm) in both gradient minimization and energy minimization was set at 0.01 kcal/mol/Å. Figure S7 shows the minimum-energy structures for the **1**–**6·C$_{60}$** complexes obtained after PM7 optimization. Several conformers were designed (and subsequently optimized) by internal rotation around the single bonds of the ester groups but only the most stable rotamers are discussed. Non-embraced host–guest arrangements, in which the crown ethers fold themselves away from C$_{60}$, and intermediate one-arm embraced conformations, in which the C$_{60}$ ball is embraced by only one arm of the exTTF-(crown ether)$_2$ receptor, were also optimized for complexes **1–3·C$_{60}$**. Figure S8 shows the optimized structures and the association energies obtained at the PM7 level for complexes **2·C$_{60}$** and **3·C$_{60}$** as representative examples. The association energy calculated for **2·C$_{60}$** at the PM7 level increases from –51.20 kcal/mol for the non-embraced conformation to –62.20 and –72.43 kcal/mol for the one-arm and two-arm embraced conformations, respectively. This indicates that both crown ether arms stabilize the complex by approximately the same energy (–10 - –11 kcal/mol). For **3·C$_{60}$**, the association energy passes from –51.56 kcal/mol for the non-embraced complex, which is similar to the value obtained for **2·C$_{60}$** (–51.20 kcal/mol), to –76.49 kcal/mol for the one-arm embraced complex. Therefore, the first arm stabilizes the complex in a larger extent (–24.93 kcal/mol) compared to **2·C$_{60}$** (–11.00 kcal/mol) due to the larger size of the crown ether and to the additional interaction with the terminal benzene ring. In contrast, the second arm stabilizes the **3·C$_{60}$** complex by a significantly lower energy of –12.26 kcal/mol due to the steric hindrance between the two crown ether arms. As a result, the final association energy obtained for the two-arm embraced **3·C$_{60}$** complex (–88.75 kcal/mol) is significantly smaller than that resulting from the sum of the energy predicted for the non-embraced complex and twice the interaction energy with the first arm (–51.56 + 2×(–24.93) = –101.42 kcal/mol).

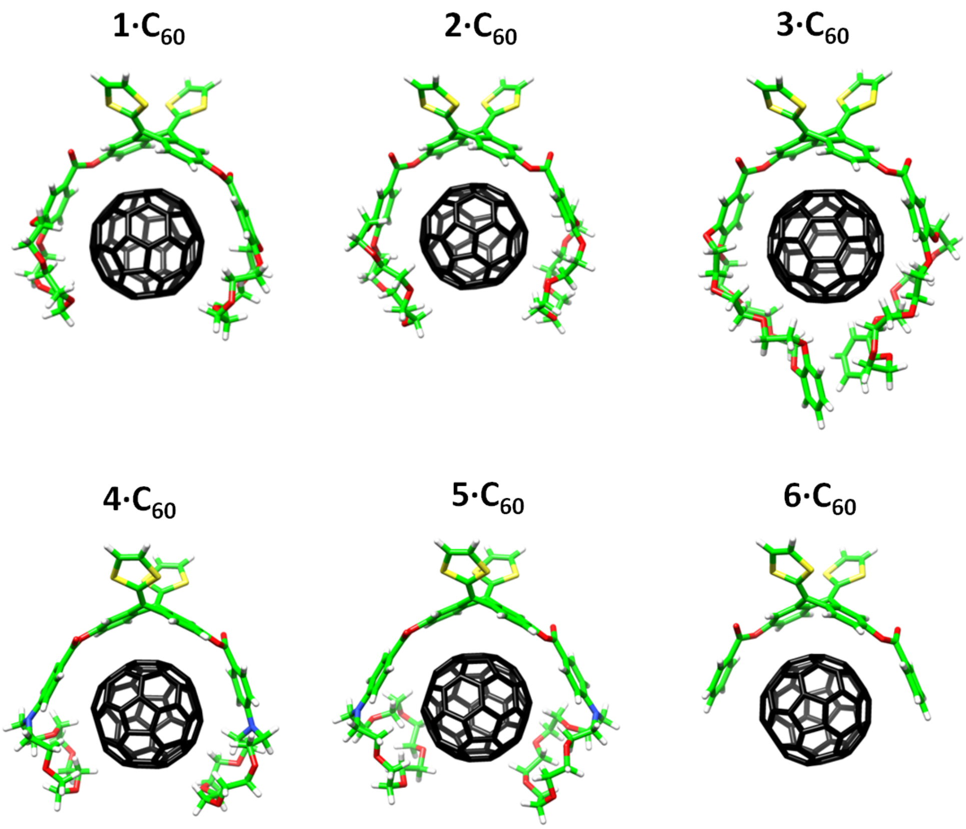

Figure S7. Minimum-energy optimized geometries calculated at the PM7 level for the supramolecular host-guest **1**-**6**·$\mathbf{C_{60}}$ complexes.

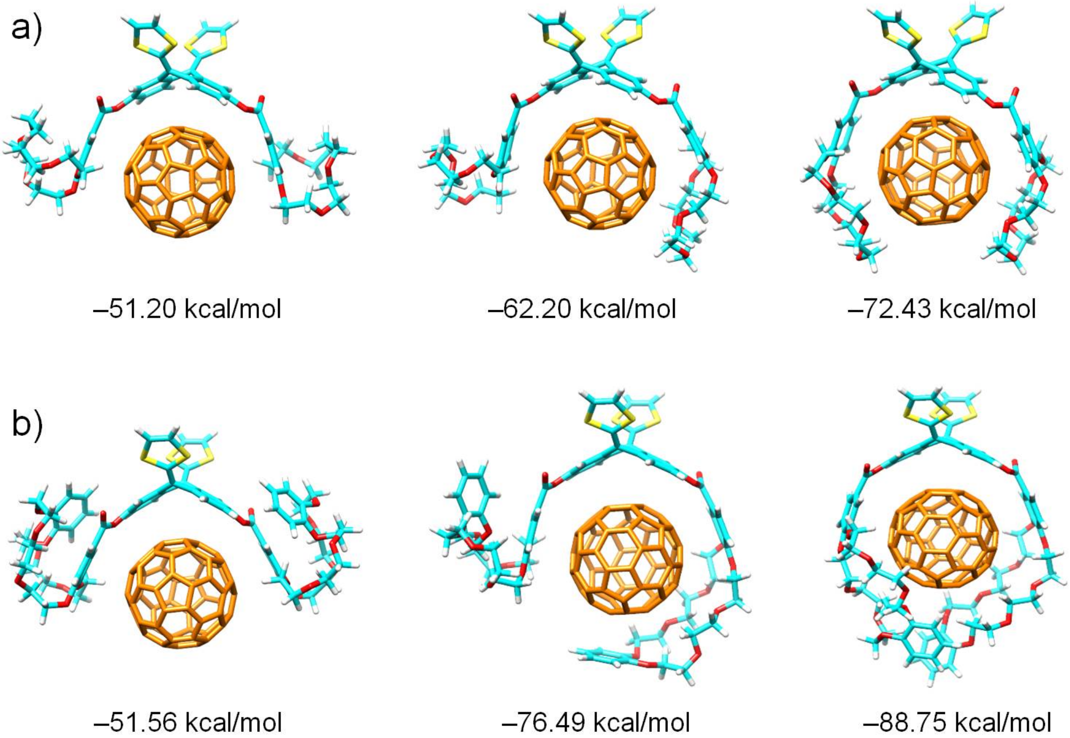

Figure S8. Minimum-energy structures and association energies computed at the PM7 level for non-embraced (left), one-arm embraced (center) and two-arm embraced (right) conformations of associates **2**·$\mathbf{C_{60}}$ (a) and **3**·$\mathbf{C_{60}}$ (b).

Accurate geometry optimizations of the supramolecular associates **1–6·C_{60}** were performed within the density functional theory (DFT) framework[12] using the B97-D Grimme's functional,[13] which includes an additional dispersion energy term, and the correlation-consistent cc-pVDZ basis set.[14] The B97-D functional is consolidated as an efficient and accurate quantum chemical approach to deal with large systems where dispersion forces are of general importance at a relative low-cost of computation.[15] Previously optimized structures at the PM7 level were used as starting geometries for the more accurate DFT optimizations. The different structural disposition adopted by the crown and aza-crown ether moieties in **2·C_{60}** and **5·C_{60}**, respectively, at the DFT minimum-energy geometries (Figure 6 in the main text) were optimized by means of the Gaussian 09 (Rev. C01) suite of programs.[16]

On the B97-D/cc-pVDZ optimized structures, the association binding energy of the complexes was estimated by single-point energy calculations using the revPBE0 correlation-exchange functional in combination with the -D3 Grimme's dispersion correction (revPBE0-D3)[12,13] and the correlation-consistent cc-pVTZ basis set.[14] The choice of the exchange-correlation functional revPBE0 is justified by its excellent performance when studying the very popular S22[17] and S66[18] non-covalent interaction databases[19] as well as when applied to other related supramolecular systems.[20] The basis set superposition error (BSSE) is expected to be negligible at the large correlation-consistent triple-ζ basis set employed and, therefore, the interaction energies are not counterpoise corrected. Moreover, note that the counterpoise method is believed to overestimate the BSSE, for which some authors propose to scale it down by half of its value.[21] The original damping function in the -D3 approach has been replaced by the Becke-Johnson damping function to provide a better performance.[22] The "resolution of identity" (RI)[23] and "chain of spheres" (COSX)[24] techniques, for the Coulomb and exchange integrals, respectively, were used to alleviate the computational cost of the more demanding steps. Note that the three-body contribution to the dispersion energy has been included because it can be significant for medium and large supramolecular systems.[25] The association energy in each associate was computed as the difference between the energy of the associate and the sum of the energies for the two constituting fragments at the geometry of the complex [E_{bind} = E(complex) – E(exTTF-tweezer) – E(C_{60})]. Geometry optimizations and single-point energy calculations at the revPBE0-D3/cc-pVTZ level were all performed using the ORCA program package (version 2.9.0).[26] Molecular orbitals (Figure S9) were plotted using the Chemcraft 1.6 software with isovalue contours of $\pm$0.03 au.[27]

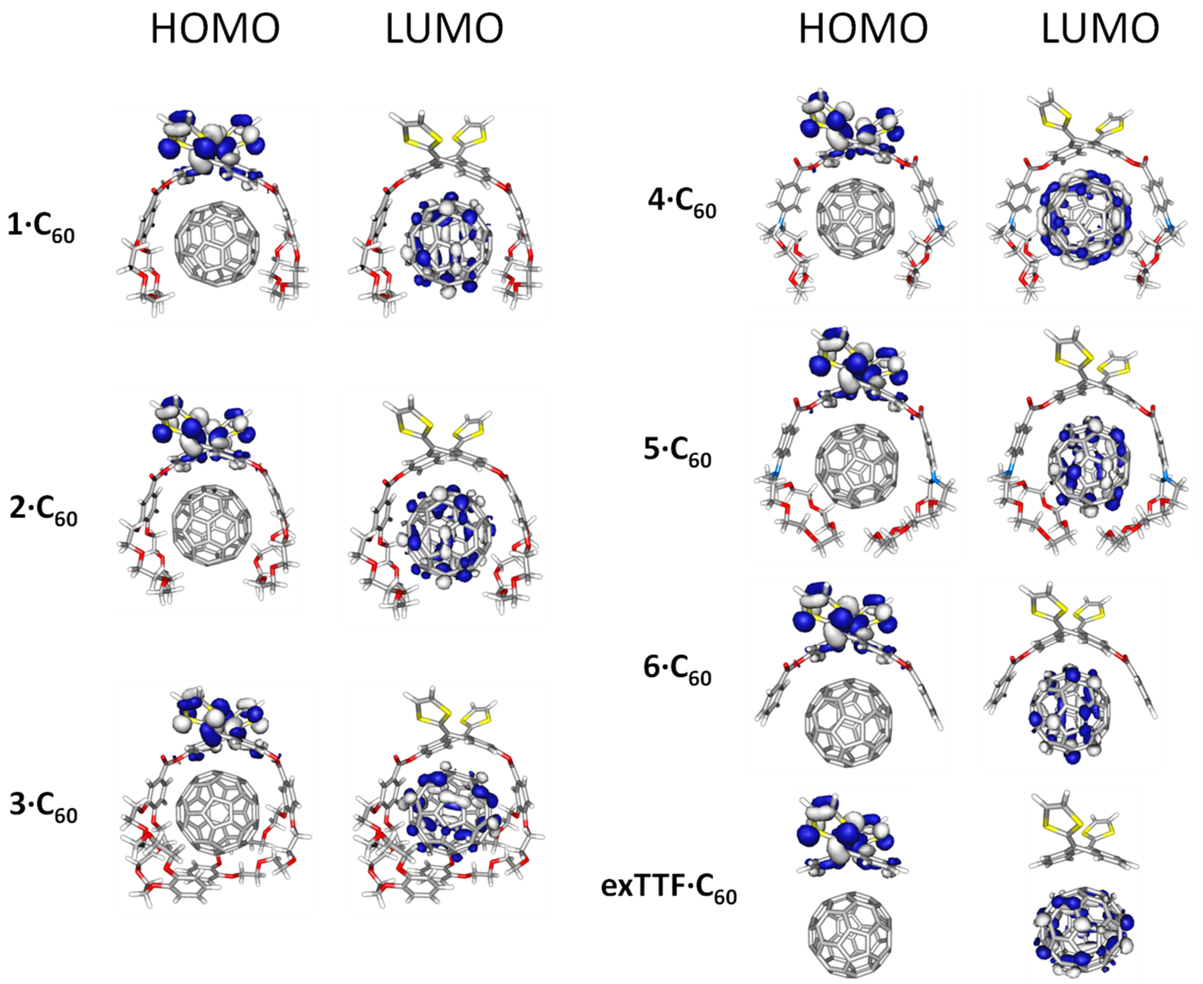

Figure S9. Isovalue contours (±0.03 au) calculated for the frontier molecular orbitals (HOMO and LUMO) of the supramolecular associates at the revPBE0-D3/cc-pVTZ level.

10. References

[1] W. L. F. Armarego, C. L. L. Chai, *Purification of Laboratory Chemicals*, Elsevier, **2003**.
[2] B. Grimm, J. Santos, B. M. Illescas, A. Muñoz, D. M. Guldi, N. Martín, *J. Am. Chem. Soc.* **2010**, *132*, 17387-17389.
[3] X.-Z. Zhu, C.-F. Chen, *J. Am. Chem. Soc.* **2005**, *127*, 13158-13159.
[4] F. Diederich, L. Echegoyen, M. Gómez-López, R. Kessinger, J. Fraser Stoddart, *J. Chem. Soc., Perkin Trans. 2* **1999**, 1577-1586.
[5] N. Yamaguchi, L. M. Hamilton, H. W. Gibson, *Angew. Chem. Int. Ed.* **1998**, *37*, 3275-3279.
[6] W. C. J. Ross, *J. Chem. Soc.* **1949**, 183-191.
[7] D. B. MacQueen, K. S. Schanze, *J. Am. Chem. Soc.* **1991**, *113*, 6108-6110.
[8] a) C. S. Wilcox, in *Frontiers in supramolecular organic chemistry and photochemistry* (Eds.: H. J. Schneider, H. Dürr), VCH, **1991**; b) G. Weber, in *Molecular biophysics* (Eds.: B. Pullman, M. Weissbluth), Academic Press, New York, **1965**.
[9] a) H. Gampp, M. Maeder, C. J. Meyer, A. D. Zuberbühler, *Talanta* **1985**, *32*, 95-101; b) H. Gampp, M. Maeder, C. J. Meyer, A. D. Zuberbühler, *Talanta* **1985**, *32*, 257-264.
[10] J. P. Stewart, *J. Mol. Model.* **2013**, *19*, 1-32.
[11] S. R. Perrin, R. M. Nicoud, in *Chiral Separation Techniques*, Wiley-VCH Verlag GmbH, **2001**, pp. 253-285.
[12] Y. Zhang, W. Yang, *Phys. Rev. Lett.* **1998**, *80*, 890-890.
[13] S. Grimme, J. Antony, S. Ehrlich, H. Krieg, *J. Chem. Phys.* **2010**, *132*, 154104-154119.
[14] J. T. H. Dunning, *J. Chem. Phys.* **1989**, *90*, 1007-1023.
[15] S. Grimme, *J. Comput. Chem.* **2006**, *27*, 1787-1799.
[16] Gaussian 09, Revision C.01,
[17] P. Jurecka, J. Sponer, J. Cerny, P. Hobza, *PCCP* **2006**, *8*, 1985-1993.
[18] J. Řezáč, K. E. Riley, P. Hobza, *J. Chem. Theory Comput.* **2011**, *7*, 2427-2438.
[19] W. Hujo, S. Grimme, *J. Chem. Theory Comput.* **2011**, *7*, 3866-3871.
[20] a) S. Grimme, W. Hujo, B. Kirchner, *Phys. Chem. Chem. Phys.* **2012**, *14*, 4875-4883; b) M. K. Rana, H. S. Koh, J. Hwang, D. J. Siegel, *J. Phys. Chem. C* **2012**, *116*, 16957-16968; c) A. Tkatchenko, O. A. von Lilienfeld, *Phys. Rev. B* **2008**, *78*, 045116; d) F. Goltl, J. Hafner, *J. Chem. Phys.* **2011**, *134*, 064102.
[21] H. Kruse, S. Grimme, *J. Chem. Phys.* **2012**, *136*, -.
[22] S. Grimme, S. Ehrlich, L. Goerigk, *J. Comput. Chem.* **2011**, *32*, 1456-1465.
[23] K. Eichkorn, O. Treutler, H. Öhm, M. Häser, R. Ahlrichs, *Chem. Phys. Lett.* **1995**, *240*, 283-290.
[24] F. Neese, F. Wennmohs, A. Hansen, U. Becker, *Chem. Phys.* **2009**, *356*, 98-109.
[25] S. Grimme, *Chem. Eur. J.* **2012**, *18*, 9955-9964.
[26] F. Neese, *WIREs Comput. Mol. Sci.* **2012**, *2*, 73-78.
[27] ChemCraft 1.6, http://www.chemcraftprog.com
